# Social Determinants of Healthcare Access: Horizontal Inequity in Rehabilitation Utilization and the Limits of Care in Mediating the Income–Depression Gradient in Türkiye

**DOI:** 10.3390/healthcare14162658

**Published:** 2026-08-21

**Authors:** Derya Azim, Muhammed Emre Güvey, Sevde Betül Kara, Sümeyra Gündem, Ecenur Aydemir, Salim Yılmaz

**Affiliations:** 1 Department of Physiotherapy and Rehabilitation, Faculty of Health Sciences, Bandırma Onyedi Eylül University, Balikesir 10200, Türkiye; dazim@bandirma.edu.tr; 2 Department of Healthcare Management, Faculty of Health Sciences, Acibadem Mehmet Ali Aydinlar University, Istanbul 34752, Türkiye; ecenur.aydemir@acibadem.edu.tr (E.A.); salim.yilmaz@acibadem.edu.tr (S.Y.); 3 Department of Physiotherapy and Rehabilitation, Faculty of Health Sciences, Istanbul Arel University, Istanbul 34010, Türkiye; betulyigit@arel.edu.tr; 4 Department of Healthcare Management, Faculty of Health Sciences, Istanbul Arel University, Istanbul 34010, Türkiye; sumeyraefe@arel.edu.tr; 5 Department of Healthcare Management, Graduate School of Health Sciences, Acibadem Mehmet Ali Aydinlar University, Istanbul 34752, Türkiye

**Keywords:** social determinants of health, health disparities, rehabilitation, healthcare access, latent profile analysis, MAIHDA, concentration index, depression, Türkiye, marginalized populations

## Abstract

**Background/Objectives:** Structural inequalities in access to healthcare persist even within systems that have achieved near-universal coverage, reflecting the enduring influence of social determinants of health on service utilization. This study examines horizontal inequity in rehabilitation and specialist care in Türkiye and investigates whether access inequality mediates the well-documented income–depression gradient. **Methods:** Analyzing the nationally representative 2022 Türkiye Health Survey (adults aged ≥15; N = 22,742), we employed Latent Profile Analysis (LPA) to construct people-centered, multidimensional bodily burden profiles, and assessed need-adjusted access using survey-weighted logistic regression, Erreygers-corrected concentration-index decomposition, Multilevel Analysis of Individual Heterogeneity and Discriminatory Accuracy (MAIHDA), and restricted cubic splines, with measurement-invariance and classification-uncertainty sensitivity analyses. Statistical mediation was examined with natural-effect models and E-value sensitivity analysis. **Results:** Although the system demonstrated responsiveness to need—78.8% of the highest-burden profile accessed specialist services—only 13.7% of this same group reached dedicated physiotherapy or rehabilitation, revealing a profound structural bottleneck in care coordination for marginalized populations with the greatest functional impairment. A persistent pro-rich gradient was confirmed by an Erreygers-corrected concentration index of 0.058 (95% CI 0.043–0.072), driven additively by income and education. Access did not mediate the income–depression pathway (natural indirect effect OR 1.001, 95% CI 1.0003–1.002). **Conclusions:** The mental health burden of low income operates through pathways that equitable healthcare access alone cannot address. These findings call for macroeconomic and people-centered health system reforms—including direct physiotherapy access, transportation subsidies, and social protection interventions—to advance health equity in rehabilitation utilization.

## 1. Introduction

Access to healthcare is not only a clinical matter; it is a profoundly social one. Across the world, structural inequalities rooted in income, education, geography, and social position shape who receives care and who does not, independently of clinical need. The concept of the social determinants of health captures this reality: factors such as poverty, educational disadvantage, and occupational insecurity are not merely correlates of poor health but constitutive forces that generate and reproduce health disparities across populations [1]. People-centered health systems are designed, in principle, to counteract these forces by ensuring that the distribution of care corresponds to the distribution of need rather than the distribution of privilege. In practice, however, the persistence of disparities even within systems that have achieved near-universal coverage reveals how deeply structural inequalities are embedded in the social fabric surrounding healthcare delivery [2,3].

Our analytic framework draws on Andersen’s Behavioral Model of Health Services Use [4], which distinguishes predisposing characteristics (age, sex, and marital status), enabling resources (income, education, and insurance coverage), and need factors as the joint determinants of utilization. Within this framework, horizontal equity requires that, conditional on need, enabling resources should not determine utilization; the persistence of income and education effects after need adjustment therefore constitutes direct evidence of inequity. This model guided the selection and classification of every variable in the analysis.

The foundational distributional challenge in health systems is captured by two complementary concepts. Vertical equity requires that those with greater need receive proportionally more care. Horizontal equity, by contrast, demands that individuals with equivalent need receive equivalent care regardless of their socioeconomic position [2,3]. A system may achieve vertical equity—directing more care to those who are sicker—while simultaneously violating horizontal equity by systematically advantaging wealthier or better-educated individuals among those with comparable need. This distinction matters because health systems can appear responsive in aggregate while masking the socioeconomic sorting that disadvantages marginalized populations within each need stratum.

The inverse care law [5] holds that the availability of good-quality care tends to vary inversely with the need of the population served. Rehabilitation and physiotherapy services are a particularly instructive domain for testing whether this law holds literally or whether a more nuanced horizontal-inequity pattern predominates: the need for these services is concentrated among older, poorer, and more functionally limited individuals, yet utilization is governed by ability to pay, health literacy, referral network access, and the organizational integration of rehabilitation within care pathways [6,7].

Türkiye offers a compelling empirical context in which to examine these questions. The Health Transformation Program, implemented progressively from the early 2000s, achieved near-universal social insurance coverage, consolidating previously fragmented schemes and dramatically reducing out-of-pocket payments for primary and secondary care [8]. This structural reform represents a significant achievement toward Universal Health Coverage (UHC) and has narrowed many dimensions of health inequality. Whether these structural gains have dissolved the socioeconomic patterning of rehabilitation access, or whether residual barriers rooted in social determinants continue to stratify utilization, remains an open empirical question. Pro-rich horizontal inequity in specialist utilization has been documented across OECD health systems and persists even under universal coverage [9,10]. Whether the same pattern extends to rehabilitation services in an upper-middle-income setting has not been established.

A further dimension of this study concerns the relationship between income and depression. The income–depression gradient is one of the most robust associations in psychiatric epidemiology, with lower-income individuals bearing a substantially disproportionate burden of depressive symptoms [11,12]. A key question is whether this gradient operates, at least in part, through barriers to rehabilitation and specialist care—that is, whether improving access would attenuate the mental health burden of low income. This hypothesis intersects with contemporary debates around the medicalization of poverty: if access fully mediates the gradient, health system reform becomes the primary policy lever; if the gradient is largely direct—operating through chronic stress, economic insecurity, and material deprivation—then macroeconomic and social protection policies become indispensable complements to health system reform. Testing this rigorously requires natural-effect models appropriate for binary mediators and binary outcomes, a methodological requirement rarely satisfied in the existing literature [13,14].

To our knowledge, no study has combined need-adjusted horizontal-inequity estimation, intersectional MAIHDA, and formal causal mediation within a single nationally representative framework for rehabilitation access in Türkiye—or, to a large extent, in any upper-middle-income country. Addressing this gap is necessary both to evaluate whether the structural gains of UHC have translated into equitable rehabilitation utilization and to determine whether access reform can plausibly serve as a mental-health policy lever. The primary objective of this study is therefore to quantify horizontal inequity in rehabilitation and specialist access relative to need; the secondary objective is to test whether access mediates the income–depression gradient. All remaining analyses—restricted cubic splines, random-forest checks, and MAIHDA decompositions—serve as supporting or sensitivity analyses for these two objectives. Based on the Andersen framework, we state two hypotheses. H1 (horizontal inequity): conditional on physical need, higher income and greater educational attainment are associated with higher utilization of rehabilitation and specialist care. H2 (mediation): part of the income–depression association is transmitted through differential access to care. To avoid circularity among need, access, and depression, the need construct was built exclusively from physical-health indicators (see Section 2.3); the causal assumptions underlying H2 are formalized in a directed acyclic graph (Figure S2).

## 2. Materials and Methods

### 2.1. Data Source and Study Population

We analyzed the public-use micro-data of the 2022 Türkiye Health Survey [15], conducted by the Turkish Statistical Institute (TurkStat) in a cross-sectional secondary-analysis design. The survey employs a stratified two-stage cluster sample with external stratification by urban/rural status, drawing 11,170 household addresses from 1117 blocks to produce nationally representative population estimates. Three questionnaires were administered covering a household roster, respondents aged 0–14, and respondents aged 15 and over. All analyses were restricted to the adult subsample (N = 22,742), as health status, functioning, service-use, and depression modules are administered exclusively to this group. Because all eligible adults within each sampled household were interviewed, the adult analytic sample (N = 22,742) exceeds the number of sampled household addresses (11,170), corresponding to a mean of approximately 2.0 interviewed adults per household. The fully observed analytic sample for multivariable models was N = 22,740, after excluding two observations for missing income data; missingness on all other derived analytic variables was negligible (≤0.01%), and complete-case analysis was used throughout.

All population estimates were weighted using the individual sampling weight (FERTFAKTOR). Because confidentiality constraints preclude release of block-level primary sampling unit (PSU) identifiers, design-based analyses used the household as the clustering unit. The intraclass correlation at the NUTS-2 regional level was modest (ICC = 1.8%), whereas the household-level ICC was substantial (29.4%), confirming the household as the dominant clustering unit. A sensitivity analysis using the 26 NUTS-2 regions as a proxy PSU was conducted to assess the impact of this design choice on standard errors. Throughout, ‘CI’ denotes the concentration index and confidence intervals are reported explicitly as ‘95% CI’.

### 2.2. Measures

#### 2.2.1. Outcome and Mediator: Access to Rehabilitation and Specialist Care

The primary outcome—and, in the mediation analysis, the mediator—was a binary indicator of access to rehabilitation or specialist care in the preceding 12 months. A broad definition coded access as present if the respondent reported any visit to a physiotherapist, physical-medicine specialist, or kinesiotherapist, or any specialist-physician contact within that period. A narrow definition was restricted to visits to physiotherapists, physical medicine specialists, or kinesiotherapists only. The broad definition served as the primary specification given its greater event count and model stability; the narrow definition was retained as a sensitivity analysis to isolate dedicated rehabilitation access. It is important to note that the broad definition encompasses all specialist-physician contacts and not only rehabilitation-specific visits.

#### 2.2.2. Depression

Depressive symptoms were measured with the eight-item Patient Health Questionnaire (PHQ-8). Item responses were recoded to the standard 0–3 metric and summed (range 0–24); the binary outcome used the conventional threshold of ≥10 for moderate-or-greater symptoms [16]. Both the binary indicator and the continuous PHQ-8 score were used in analyses, the latter as a sensitivity specification.

#### 2.2.3. Disability and Bodily Need Indicators

Functional difficulty was assessed with the Washington Group short set; following its standard methodology, disability was operationalized as a binary indicator (serious difficulty in ≥1 domain) and a domain-count index rather than a latent factor [17]. Five bodily need indicators were constructed: a musculoskeletal burden count (arthrosis, low-back, and neck problems), a bodily pain score (reverse-coded so that higher values denote more pain), the Washington Group disability-domain count, self-rated general health (higher = worse), and a summed weekly activity frequency score across four activity domains (walking, cycling, sport, and muscle strengthening; range 0–28) [18].

#### 2.2.4. Socioeconomic Position and Covariates

Household income was measured using the 20-band ordered measure of monthly net household income (HANE_GELIR_GRUP). Education was recoded from the ISCED-style survey item into four ordered levels (none/low; primary–lower-secondary; upper-secondary; tertiary), with ‘unknown’ set to missing. Additional covariates included sex, single-year age, marital status, employment status, and social security coverage status.

### 2.3. Analytic Strategy

Analyses followed a chained design in which the output of each step served as input to the next. Unless otherwise specified, estimation was performed in R (version 4.6.0).

Step 1—Measurement Validation. Confirmatory factor analysis (CFA) with the WLSMV estimator assessed the dimensionality of the PHQ-8 and the Washington Group short set, treating items as ordinal. Model fit was evaluated using CFI, TLI, RMSEA, and SRMR, with standardized loadings, Cronbach’s α, and McDonald’s ω reported [19]. Measurement invariance of the PHQ-8 across income tertiles, education groups, and sex was tested with multi-group ordinal CFA under the Wu–Estabrook identification [20], comparing configural, threshold-constrained, and threshold-and-loading-constrained models. Invariance was judged by ΔCFI ≥ −0.010 and ΔRMSEA ≤ +0.015 [21,22], as scaled χ^2^ difference tests are oversensitive at this sample size. For the education grouping, a sparse top-category cell required collapsing response categories 2–3. Invariance is a precondition for the group contrasts that follow: when measurement parameters differ across groups, the group-specific artifact propagates into the structural estimates and inflates their apparent between-group dispersion, so that part of an observed socioeconomic gradient may be measurement-induced rather than substantive [23]. Where invariance does fail, stabilizer-variable methods offer one route to absorbing the artifactual component [23]; that approach was not required here, because full threshold-and-loading invariance was supported for every grouping tested.

Step 2—Latent Profile Analysis (LPA). To move beyond simple disease counts toward a people-centered, multidimensional characterization of bodily burden, LPA (Gaussian finite-mixture models, mclust package version 6.1.1) was estimated on the five bodily need indicators. Depression and healthcare access were excluded from the profile construction by design—a prespecified safeguard we term the Circularity Shield. If the need construct used to standardize access included variables that are themselves outcomes or mediators of interest, the resulting inequity estimates would be biased and the independence assumptions required for both the horizontal-inequity analysis and the mediation chain would collapse; building need exclusively from physical health indicators anchors the need-adjusted inequity index in demonstrably independent clinical information. LPA identifies subgroups sharing similar patterns of physical need across multiple domains simultaneously, capturing the co-occurrence and severity of musculoskeletal burden, pain, functional limitation, and self-rated health in ways that a single-symptom count cannot. Models with one to six profiles and four covariance parameterizations were compared using the BIC, entropy, smallest-class size, and interpretability criteria [24,25]. The average posterior probability (AvePP) matrix and posterior-probability distributions were examined to characterize classification quality. Robustness of downstream estimates to classification uncertainty was assessed with two complementary approaches: proportional assignment, weighting individuals by their posterior probabilities, and pseudo-class draws, in which profile membership was repeatedly sampled (K = 50) from each individual’s posterior distribution, the access model re-estimated per draw, and estimates pooled with Rubin’s rules, reporting the fraction of missing information (FMI) [26,27].

Step 3—Access Models. (a) Survey-weighted logistic regression (svyglm [28]; FERTFAKTOR weights and household clustering) modeled broad access against income, education, and covariates, with need adjusted using continuous scores (primary) and latent profiles in parallel specifications. Odds ratios (ORs) with 95% CI, variance inflation factors, likelihood ratio tests, and McFadden’s pseudo-R^2^ are reported. (b) The Erreygers-corrected concentration index was the primary measure of income-related inequality, with the standard concentration index (CI) reported alongside; both were computed analytically and cross-checked with the rineq package version 0.3.0. A Wagstaff-type decomposition apportioned the index into contributions from socioeconomic, need, and demographic factors; the need-standardized horizontal inequity index (HI) was read as the non-need socioeconomic share of the decomposition [2,3,29]. (c) MAIHDA assigned individuals to 36 intersectional strata (sex × income tertile × age group × education group); two-level logistic models yielded the variance partition coefficient (VPC) and proportional change in variance (PCV) after adding main effects (lme4 package version 1.1-37) [30,31,32,33]. Consistent with standard MAIHDA practice, these two-level models were estimated without survey weights; all other regression, cross-tabulation and concentration index estimates are survey-weighted. MAIHDA is particularly well-suited for health equity research because it simultaneously models additive and interactive effects of multiple social identities, allowing detection of whether population-level inequalities reflect general socioeconomic gradients or emerge at specific intersections of disadvantage. (d) Restricted cubic splines (df = 3) were used within the survey-weighted logistic framework to assess departure from linearity in the income–access relationship.

Step 4—Statistical Mediation. The income → access → depression pathway was examined with natural-effect models (medflex package version 0.6-10) and a regression-based approach incorporating an exposure–mediator interaction and E-value sensitivity analysis (CMAverse package version 0.1.0), both adjusting for bodily need and demographic confounders [13,14,34,35,36]. Natural direct and indirect effects (NDE, NIE) are reported on the odds-ratio scale. Because the survey data are cross-sectional, the temporal ordering of income, access, and depression is assumed rather than observed; the mediation analysis is therefore associational and describes the statistical decomposition of the income–depression gradient under stated causal assumptions formalized in a directed acyclic graph (DAG; Figure S2). Natural-effect models were estimated without survey weights, as the medflex implementation does not accommodate design-based weighting.

Sex (a biological attribute as recorded by the survey) was included as a covariate and examined descriptively; the survey did not capture gender identity separately. The analysis additionally used the lavaan version 0.6-21 [37], psych version 2.6.5 [38], rineq version 0.3.0 [39], lme4 version 1.1-37 [40], car version 3.1-3 [41], ranger version 0.16.0 [42], pdp version 0.8.1 [43], ggplot2 version 3.5.1 [44] and patchwork version 1.3.0 [45] packages; full package versions and purposes are listed in Table S21.

## 3. Results

Among 22,742 adults aged ≥ 15, the prevalence of moderate-or-greater depressive symptoms (PHQ-8 ≥ 10) was 4.5%, and the prevalence of disability (serious difficulty in ≥1 Washington Group domain) was 13.6%. Broad access to rehabilitation or specialist care in the preceding 12 months was reported by 62.5% of the sample, whereas physiotherapy-specific access was far less common at 5.6%. The analytic sample for multivariable models was N = 22,740 (Table 1).

### 3.1. Measurement Validation

A one-factor PHQ-8 model demonstrated excellent fit (CFI = 0.980, TLI = 0.973, SRMR = 0.041; standardized loadings 0.80–0.89; Cronbach’s α = 0.881, McDonald’s ω = 0.949). The elevated scaled RMSEA (0.093) reflects the known upward bias of this index at low degrees of freedom in very large samples [19], rather than structural misfit; the PHQ-8 depression index was therefore retained as a summed score. The Washington Group model showed acceptable incremental fit (CFI/TLI 0.980/0.973) but poor absolute fit (SRMR = 0.166, RMSEA = 0.123), with markedly heterogeneous loadings (vision 0.54 to hearing 0.98), consistent with distinct functioning domains rather than a single latent trait. Disability was accordingly operationalized as a domain-count index and binary indicator rather than as a latent factor.

Measurement invariance of the PHQ-8 was supported across income tertiles, education groups, and sex: constraining thresholds and factor loadings to equality produced no meaningful deterioration in fit at any step (all ΔCFI ≥ −0.002 and all ΔRMSEA ≤ 0; Table S4). PHQ-8 scores can therefore be compared across the socioeconomic and demographic groups central to this study, supporting the use of the summed score and the ≥10 threshold along the income and education gradients.

### 3.2. Latent Profile Analysis: Mapping People-Centered Bodily Burden

**Figure 1:**
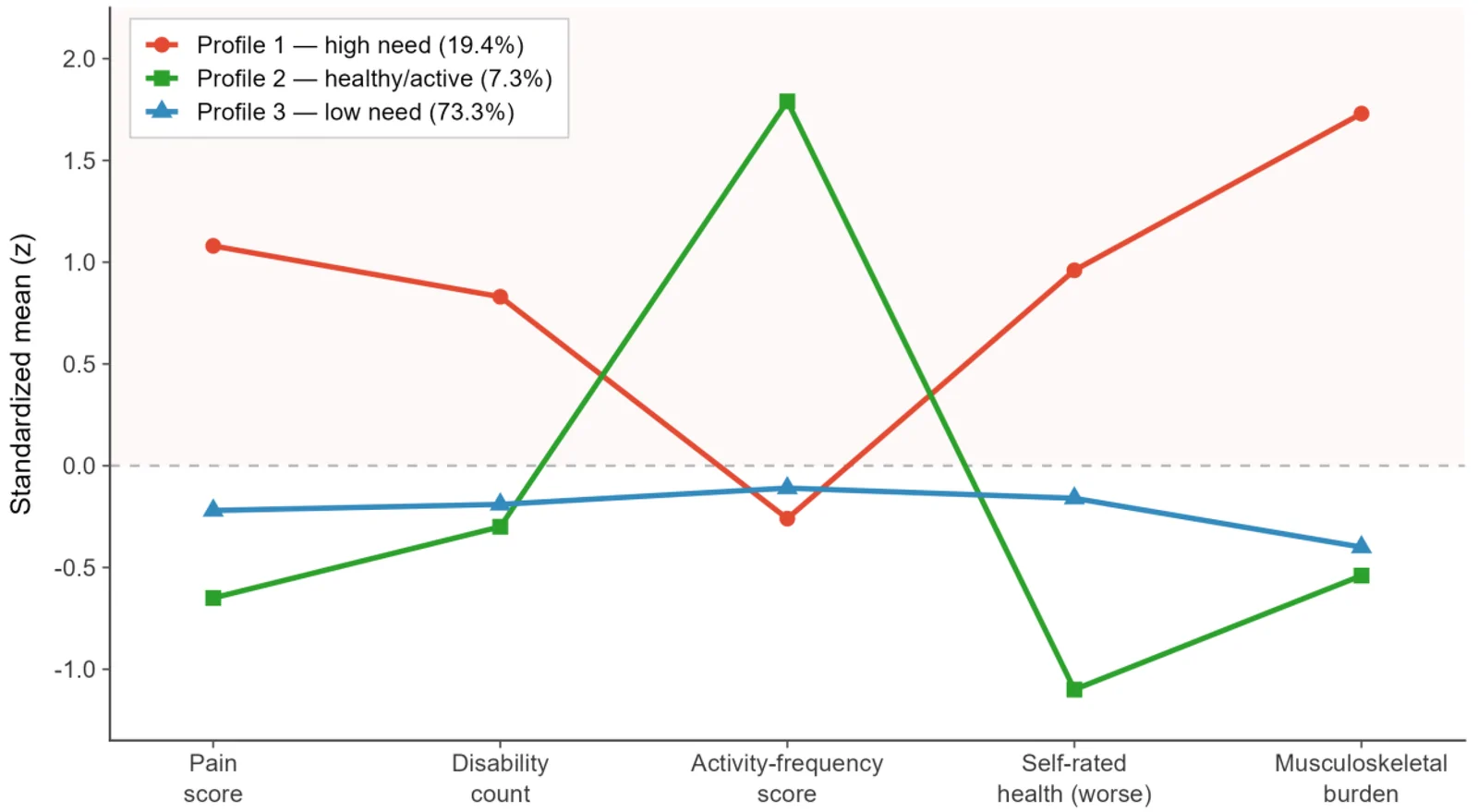
Standardized (z-score) mean of each bodily need indicator by latent profile: high need (Profile 1, 19.4%), healthy/active (Profile 2, 7.3%), and low need (Profile 3, 73.3%). Higher values on self-rated health indicate worse health. The activity frequency score sums the number of days per week reported separately for four domains—walking ≥ 10 min, cycling, sport, and muscle strengthening activities—and therefore ranges from 0 to 28 rather than 0 to 7. The Circularity Shield specifies that depression and access indicators were excluded from the LPA by design.

Latent profile analysis on the five bodily need indicators yielded a three-profile, class-invariant diagonal-covariance (EEI) solution, selected on the joint basis of the BIC elbow, classification quality, minimum class size, and substantive interpretability (full fit statistics in Table S5). The three profiles comprised: a High-Need group (Profile 1, 19.4%), characterized by the highest musculoskeletal burden, pain severity, disability-domain count, and poor self-rated health; a Healthy/Active group (Profile 2, 7.3%), with the lowest overall burden and the highest physical activity; and a Low-Need/General Population group (Profile 3, 73.3%). Standardized indicator means are displayed in Figure 1.

The entropy of the three-profile solution was 0.68; the average posterior probability matrix (Table S6) showed near-perfect classification for the high-need profile (AvePP = 0.97), adequate classification for the low-need profile (0.83), and lower precision at the boundary between the two lower-need groups (healthy/active AvePP = 0.69). Two sensitivity analyses confirmed that classification uncertainty does not bias the main findings. A proportional assignment analysis weighting individuals by posterior probabilities yielded identical income odds ratios (1.021, 95% CI 1.016–1.025), and a pseudo-class draws analysis propagating classification uncertainty fully into the standard errors (K = 50 draws, Rubin’s rules) produced a pooled income odds ratio of 1.021 (95% CI 1.012–1.029) with a fraction of missing information of only 0.3% for income and below 0.4% for all education coefficients (Table S11). Classification uncertainty was concentrated in the contrast between the two low-burden profiles (FMI = 45.5% for the healthy/active profile, AvePP = 0.69), precisely where the AvePP matrix indicated boundary imprecision. Uncertainty was not absent elsewhere: the high-need profile carried an FMI of 10.8%, an order of magnitude above any socioeconomic coefficient. Its estimate nevertheless remained stable (pooled OR 2.40, 95% CI 2.18–2.65, vs. 2.44 under modal assignment), so the substantive finding does not depend on how boundary cases are assigned.

### 3.3. Need-Adjusted Access and Horizontal Inequity

**Figure 2:**
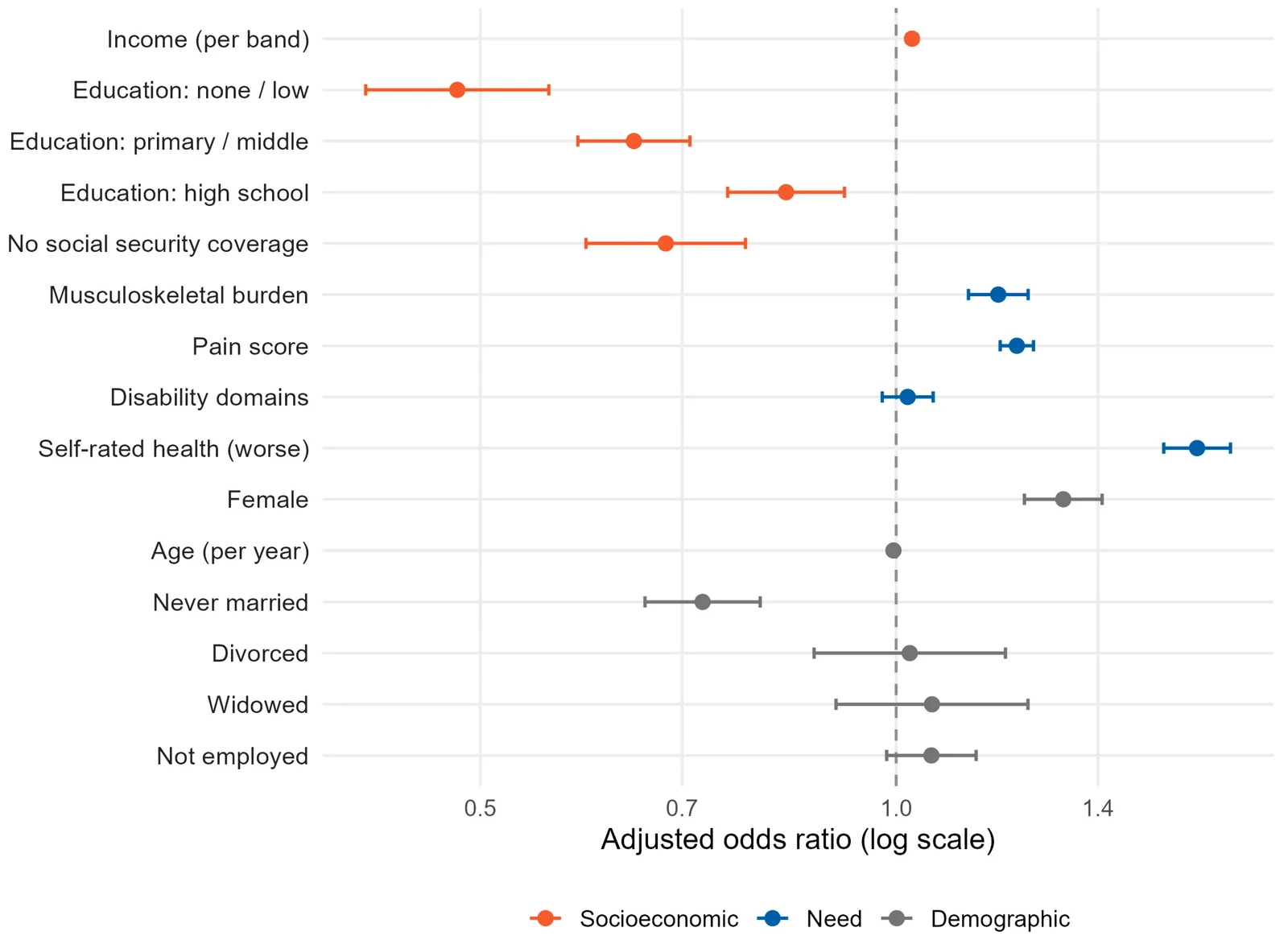
Need-adjusted determinants of broad access (primary survey-weighted logistic model; Model A). Points are adjusted odds ratios; bars are 95% confidence intervals. Color denotes factor category: red = socioeconomic; blue = need; grey = demographic. Reference categories: education = tertiary; sex = male; marital status = married; employment = employed; social security coverage = covered. N = 22,740.

In survey-weighted logistic models with need held constant (Figure 2), income retained an independent, positive association with broad access. The per-band income odds ratio was 1.017 (95% CI 1.009–1.025) without need adjustment, 1.027 (1.018–1.035) after adjustment by continuous need scores (primary model), and 1.021 (1.013–1.029) after adjustment by latent need profile (all *p* < 0.001). The slight strengthening of the income association after need adjustment reflects a suppression pattern: need is concentrated among lower-income individuals and partially masks the pro-rich gradient. Education showed a markedly steeper association, with those with no or low education having roughly half the odds of access compared with tertiary-educated peers (OR 0.48, 95% CI 0.41–0.56). Bodily need indicators behaved as expected in a need-responsive system, with the high-need profile showing substantially elevated odds of access (OR 2.44, 95% CI 2.22–2.68; Model B). All variance inflation factors were below 2.3, indicating negligible multicollinearity (full model coefficients in Table S8).

A sensitivity analysis using the 26 NUTS-2 regions as proxy PSUs confirmed that the income association remained statistically and substantively significant under the more conservative design assumption (income OR 1.027, 95% CI 1.012–1.042; CI width ratio NUTS-2/household = 1.76).

### 3.4. Concentration Index Decomposition

**Figure 3:**
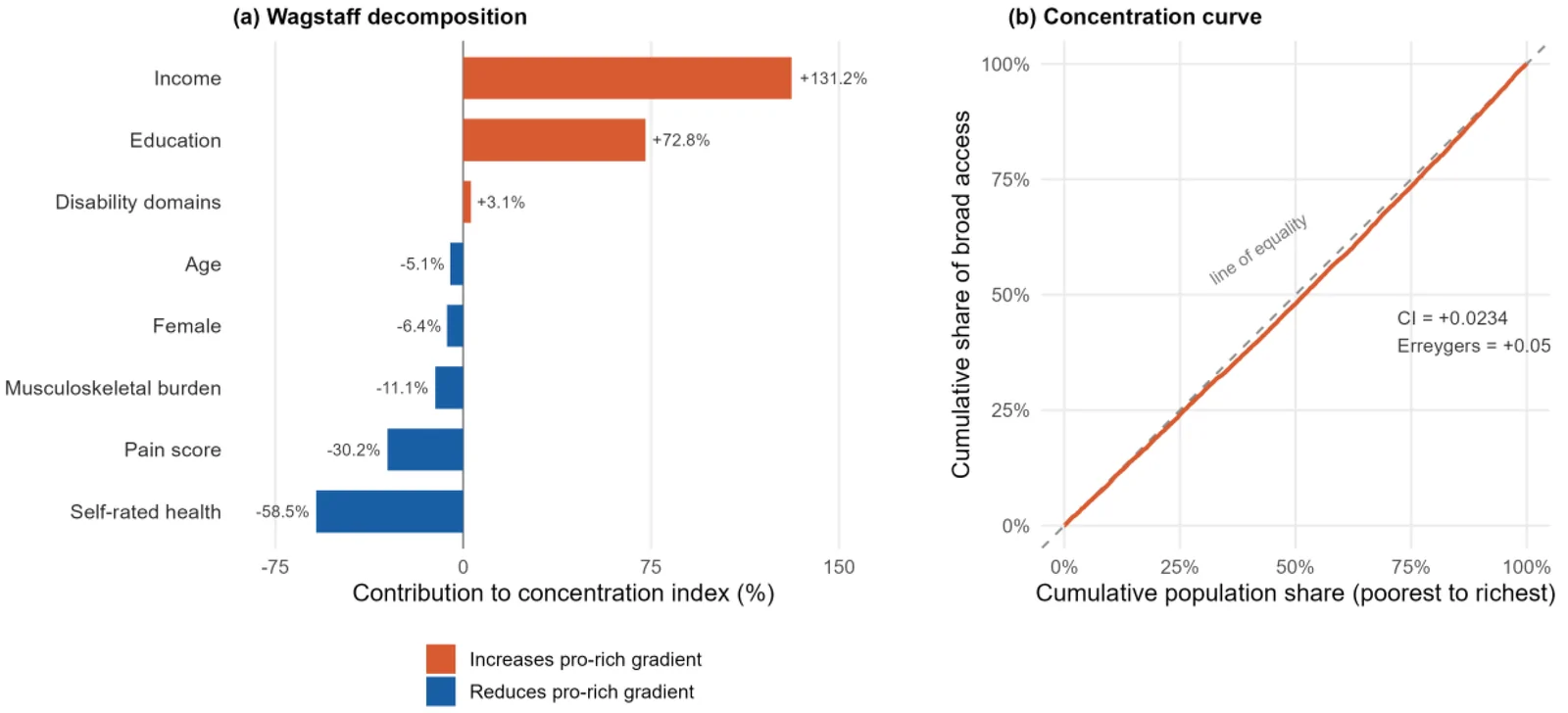
Income-related inequity in broad access to rehabilitation and specialist care. (**a**) Wagstaff decomposition of the concentration index: bars show each factor’s contribution as a percentage of the standard concentration index (CI = +0.0234), with red indicating factors that increase the pro-rich gradient and blue indicating factors that reduce it (need variables). (**b**) Concentration curve for broad access by income rank: the curve lies below the line of equality, indicating a pro-rich distribution (standard CI = +0.0234; Erreygers-corrected CI = +0.058). Horizontal axis: cumulative population share ranked from poorest to richest; vertical axis: cumulative share of access.

The Erreygers-corrected concentration index for broad access was +0.058 (95% CI 0.043–0.072), and the standard index was +0.023 (95% CI 0.018–0.029), both indicating a pro-rich distribution (Figure 3b). For narrow physiotherapy-specific access, the Erreygers index was +0.004 and the standard index was +0.018. These two figures should not be read as indicating greater equity in rehabilitation-specific access: the Erreygers correction multiplies the standard index by 4μ, and because narrow-access prevalence (5.6%) is an order of magnitude below broad-access prevalence (62.5%), the correction shrinks the narrow-access index far more. On the model-based scale the narrow-access income gradient is in fact the steeper of the two (OR 1.027 vs. 1.021; Table S12). The need-standardized horizontal inequity index exceeded the gross index on the Erreygers scale (+0.118 versus +0.058), confirming that standardizing for need amplifies measured inequity: because need is concentrated among lower-income individuals, unadjusted access rates understate the true socioeconomic gradient in care relative to equal need.

Wagstaff decomposition (Figure 3a) showed that socioeconomic factors contributed +0.1175 to the Erreygers index (income +0.0756 and education +0.0419) against a total index of +0.058, while need factors contributed −0.0558 and demographics −0.0066. Expressed as percentages of the total, these contributions exceed 100% because the offsetting need contribution leaves a small denominator; the absolute contributions above are the interpretable quantities. In this decomposition, socioeconomic factors accounted for essentially all of the pro-rich gradient, with the distribution of physical need offsetting rather than driving it. Because the Erreygers correction rescales the standard index by a constant, percentage contributions are invariant to the choice of index.

### 3.5. Intersectional Analysis: MAIHDA Findings

Across 36 intersectional strata defined by sex, income tertile, age group, and education, the null-model variance partition coefficient was 3.8% (between-stratum variance 0.1288), indicating that only a small share of variation in access lies between intersectional strata. After adding main socioeconomic effects, stratum-level variance fell to near zero (0.0036), representing a proportional change in variance of 97.2% and leaving a residual interaction component of only 2.8% (stratum-level predicted access in Table S14).

These findings indicate that disadvantages in access operate primarily through broadly acting social gradients rather than through highly specific intersectional interactions. The gradient across strata is gradual and predominantly additive: low income and lower education reduce access across multiple demographic positions, and no isolated, severely disadvantaged cluster was identified.

### 3.6. Access by Need Profile and Income Tertile

Cross-tabulation of weighted access rates by latent need profile and income tertile confirms both vertical responsiveness and horizontal inequity. Broad access increased monotonically with bodily need: 78.8% in the high-need profile, 58.4% in the low-need profile, and 48.7% in the healthy/active profile (Rao–Scott *p* < 0.001). Access was thus positively and monotonically associated with bodily need.

However, the most revealing finding emerges when examining the type of service accessed. Among the highest-need individuals—those with the greatest musculoskeletal burden, pain, disability, and poor self-rated health—only 13.7% accessed dedicated physiotherapy or rehabilitation services, compared with 78.8% broad specialist contact in the same group. The gap between entering the healthcare system and receiving rehabilitation-oriented care therefore exceeded 60 percentage points in the highest-need profile. For narrow rehabilitation access, the contrast is even starker: 13.7% in the high-need profile versus 3.6% and 2.6% in the lower-need profiles (*p* < 0.001).

Within each need profile, access rose steadily with income, confirming horizontal inequity at every level of bodily burden (Table 2). In the high-need profile, the gap between the lowest and highest income tertile was 4.1 percentage points (77.3% vs. 81.4%); in the low-need profile, this gap widened to 10.3 percentage points (53.3% vs. 63.6%). The income gradient was smallest—and partially attenuated by ceiling effects—among those with the greatest need, and widest among those with lower or more discretionary needs.

### 3.7. Functional Form of the Income–Access Gradient

A restricted cubic spline (df = 3) within the survey-weighted logistic framework provided no evidence of departure from linearity (Wald test on the non-linear basis terms: F = 1.40, df = 2 and 10,009, *p* = 0.246; the four-degree-of-freedom specification gave F = 1.25, df = 3 and 10,008, *p* = 0.290). An unweighted likelihood-ratio test indicated a marginal improvement in fit (*p* = 0.014), but this test does not account for the complex survey design. Predicted access curves from the linear and spline specifications overlapped closely (Figure S1), and the spline revealed a modest plateau across the lower half of the income distribution followed by a steady rise above the median—consistent with the partial-dependence profile from supplementary random-forest analysis (Table S16, linear-fit R^2^ = 0.92). The design-based tests therefore supported the linear specification, which was retained for both parsimony and statistical adequacy.

### 3.8. Mediation: Does Access Explain the Income–Depression Gradient?

Two mediation estimators agreed on the structure of the income–depression association. The natural direct effect of income on depression was protective and statistically significant (medflex NDE OR 0.975, 95% CI 0.961–0.988; CMAverse pure NDE OR 0.979, *p* < 0.001). The natural indirect effect through access was negligible (medflex NIE OR 1.001, 95% CI 1.0003–1.002; CMAverse pure NIE OR 1.003, 95% CI 0.999–1.008, *p* = 0.30) (Tables S18 and S19). Although the very large sample rendered the medflex indirect effect technically distinguishable from zero, its magnitude was trivial—approximately 1.02 across the full income range—and the second estimator placed the indirect effect well within sampling noise.

The mediator–outcome path was imprecise but, if anything, positive (access associated with more depression, OR ≈ 1.48, *p* = 0.06 after adjustment), consistent with confounding by indication rather than a protective pathway. The E-value for the direct effect was 1.17, and the indirect effect’s confidence interval crossed the null. A continuous PHQ-8 sensitivity analysis reproduced the pattern. The income–depression gradient is therefore essentially direct in this cross-sectional decomposition and is not statistically transmitted through rehabilitation or specialist care access.

## 4. Discussion

### 4.1. Vertical Responsiveness Within a Horizontal Inequity Framework

This study demonstrates that Türkiye’s healthcare system has achieved meaningful vertical equity in access to specialist services: those with the greatest bodily burden are the most likely to access care, with 78.8% of the highest-need profile reporting specialist contact in the preceding year. This is a genuine achievement of the Health Transformation Program and near-universal coverage, and it stands in contrast to the absolute exclusion described by the inverse care law in its original formulation [5]. However, the more salient equity challenge in Türkiye is not one of absolute exclusion but of horizontal inequity within need strata: among individuals with equivalent bodily burden, those with higher income and greater educational attainment receive systematically more care. This is consistent with international evidence showing that lower-income and sicker adults in OECD countries report poorer healthcare access experiences, even when insured [46], and with findings from other upper-middle-income settings in which formal coverage has not eliminated socioeconomic patterning of utilization [47].

The direction and magnitude of the gradient observed here are not exceptional in international perspective: van Doorslaer and colleagues documented pro-rich horizontal inequity in specialist utilization in virtually all of the 21 OECD countries they examined [9], and an updated analysis of 18 OECD countries confirmed that, for equal need, higher-income individuals remain more likely to consult a doctor [10]. Direct numerical comparison requires care, because our headline horizontal inequity index is Erreygers-corrected (+0.118), whereas the OECD analyses report concentration indices of need-standardized use; on that scale our directly need-standardized index is +0.044 (Table S13). The comparison that can be drawn with confidence is directional rather than ordinal: pro-rich inequity in specialist contact was present in every country van Doorslaer and colleagues examined, and in all but two of the countries in the later analysis, indicating a broader phenomenon in which universal coverage coexists with socioeconomically graded specialist and rehabilitative care.

The decomposition results are particularly revealing about the social determinants of this inequity. Socioeconomic factors—primarily income and education—account for approximately 204% of the concentration index, while the distribution of physical need offsets approximately 97%. Put differently, the pro-rich gradient in access is entirely generated by socioeconomic forces and would be even larger if need were not disproportionately concentrated among lower-income groups. Income and education each make large, additive contributions to inequity, and these effects are not concentrated in a few isolated subgroups but operate broadly across the population, as confirmed by the MAIHDA results. This pattern calls for broad equity-oriented health system reforms targeting the general mechanisms by which socioeconomic position shapes rehabilitation utilization—including co-payments, transportation costs, time constraints, referral literacy, and health literacy—rather than narrowly targeted programs for specific intersectional subgroups alone. Notably, the income gradient within need strata was smallest—and partially attenuated by ceiling effects—among those with the greatest need, and widest among those with lower or more discretionary needs, suggesting that socioeconomic sorting governs more discretionary forms of utilization more powerfully.

### 4.2. The Structural Bottleneck: From System Entry to Rehabilitation

A revealing tension is apparent already at the descriptive level: broad specialist contact is common, yet dedicated rehabilitation—the service most directly aligned with musculoskeletal and functional need—reaches very few. The most striking finding of this study is not the socioeconomic gradient in specialist access per se, but the structural gap between broad specialist contact and rehabilitation-specific utilization. Among individuals in the highest-need profile, 78.8% accessed specialist care but only 13.7% received dedicated physiotherapy or rehabilitation. This more than 60 percentage point gap reveals a profound structural bottleneck in the care pathway: the healthcare system is capable of reaching individuals with high physical need at the point of specialist contact, yet it systematically fails to translate that contact into rehabilitation-oriented, people-centered care.

This gap reflects not simply a shortage of rehabilitation resources but a fragmentation of care pathways that is well documented in the international literature. Rehabilitation depends not only on patient need but on how physiotherapy is positioned within care pathways, the referral practices of primary and specialist physicians, the geographic availability of services, and the extent to which rehabilitation is embedded in routine clinical protocols for musculoskeletal and disabling conditions [48,49,50,51]. In Türkiye, rehabilitation services have traditionally relied on physician-led referral pathways, which means that even patients who enter the specialist system may not be directed to physiotherapy unless the referring physician recognizes rehabilitation need, has access to an appropriate referral network, and the patient can navigate the subsequent administrative and logistical steps. Direct access to physiotherapy—where patients can self-refer without a physician referral—has been shown to reduce the burden on primary care, decrease unnecessary investigations, and maintain equivalent clinical outcomes [49,52]; however, implementation evidence remains limited and context specific [53,54].

For marginalized populations with the greatest functional impairment, these structural barriers accumulate. Transportation costs, the time cost of multiple care episodes, the complexity of navigating referral networks, and the out-of-pocket costs of rehabilitation sessions—which may not be fully covered even under universal insurance—compound the disadvantage of lower income and lower health literacy. Recent Ministry of Health initiatives to expand Healthy Life Centers and embed physiotherapists within community-based preventive and supportive services represent a promising structural response to this bottleneck, potentially enabling direct access to rehabilitation for communities that would otherwise face multiple systemic barriers [55].

### 4.3. The Null Mediation Finding and the Limits of Healthcare Access

A central finding of this study is that access to rehabilitation and specialist care does not statistically mediate the income–depression gradient. The natural indirect effect through access was negligible across both mediation estimators and was not robust in either magnitude or direction, with a tendency toward positive associations between access and depression consistent with confounding by indication. The direct effect of income on depression—protective, substantial, and robust—accounts for virtually all of the associations observed in the data.

This null mediation finding has important implications for the debate on the medicalization of poverty. The income–depression gradient is frequently invoked to justify healthcare investment as a mental health intervention: if access to care mediates the income–depression relationship, then improving access becomes a lever for reducing the disproportionate mental health burden borne by low-income and marginalized populations. Our findings challenge this framing. In the Turkish context, the mental health costs of low income appear to operate primarily through the direct pathways associated with chronic economic insecurity, material deprivation, and the psychosocial stress of poverty—pathways that are not substantially modified by access to specialist or rehabilitation care as measured in this study [11,12]. This is consistent with a growing body of evidence that social determinants of mental health, including housing instability, food insecurity, occupational precarity, and neighborhood disadvantage, generate depression burden through mechanisms that healthcare access cannot address [56,57,58]. This pattern maps directly onto the WHO Commission’s distinction between structural determinants of health inequities—income distribution, labor-market position, and welfare and housing policy—and the intermediary determinants (material circumstances and psychosocial stress) through which they operate [59]: our findings indicate that the income–depression gradient is not transmitted through the healthcare component of the intermediary determinants, and therefore implicate the material and psychosocial intermediary pathways through which structural determinants operate.

This does not imply that improving rehabilitation access has no value; equitable access to care is intrinsically important for functional health, quality of life, and the performance of a people-centered health system. However, the present findings suggest that improving access to rehabilitation and specialist care, while a necessary component of health equity policy, is unlikely by itself to substantially attenuate the mental health burden associated with socioeconomic disadvantage. Addressing that burden requires macroeconomic and social protection policies—employment security, social housing, income support, food security programs, and community engagement initiatives—that target the upstream social determinants of depression directly.

### 4.4. The Circularity Shield: Methodological Implications for Health Equity Research

A methodological contribution of this study is the operationalization of the Circularity Shield in need-standardized inequity analysis. By constructing the bodily need construct exclusively from physical health indicators and excluding depression and access by design, the study ensures that the horizontal inequity index captures genuine need-adjusted socioeconomic differences in utilization rather than conflating need with outcomes. This has practical implications for health equity measurement: incorporating depression into the need construct would collapse the independence assumptions required for both horizontal inequity analysis and the mediation chain, producing estimates that confound the measurement of inequity with the very pathways under investigation. The trade-off is that the need construct does not capture mental-health need, and individuals with depression but no prominent physical burden may have their specialist visits (including mental health contacts included in the broad access definition) classified as discretionary rather than need-driven. This may modestly overstate the pro-rich horizontal inequity index for mental health-related specialist utilization; however, this limitation is preferable to the circularity that would arise from incorporating mental health need into the construct.

### 4.5. Limitations

Several limitations should be noted. The cross-sectional design fixes the income–access–depression ordering only by theoretical assumption, formalized in the DAG (Figure S2), rather than by observation; the mediation analysis is therefore associational and not causal. A further limitation is that the natural-effect models could not incorporate the survey weights or the clustered sampling structure; the mediation estimates should therefore be read as associational within-sample results rather than design-based population estimates. Block-level PSU identifiers were withheld from the microdata for confidentiality reasons; a NUTS-2 proxy-PSU sensitivity analysis widened confidence intervals (income OR 95% CI from [1.018, 1.035] to [1.012, 1.042]) but preserved all substantive conclusions. The latent-profile entropy was moderate (0.68) overall, although the high-need profile driving the main findings showed near-perfect classification (AvePP = 0.97), and both proportional assignment and pseudo-class draws analyses yielded essentially identical socioeconomic associations, with the fraction of missing information below 0.4% for all income and education coefficients (Table S11). Restricted cubic splines provided no evidence of departure from linearity in design-based tests (*p* = 0.246), and predicted access curves from the linear and spline specifications overlapped closely, supporting the retention of the linear specification. Self-reported twelve-month utilization is subject to recall bias, and the mediator–outcome path remains open to confounding by indication. The broad access definition includes all specialist-physician contacts and not only rehabilitation-specific visits, which may dilute estimates for the rehabilitation-specific gradient; the narrow-access sensitivity analysis addresses this but with reduced statistical precision.

A further conceptual limitation concerns the operationalization of access. Following Levesque and colleagues’ multidimensional framework [60], utilization captures only the realized endpoint of access and does not measure approachability, availability and accommodation, acceptability, affordability in full, or the appropriateness and quality of care received; nor does contact with services equate to effective coverage in Tanahashi’s sense [61]. Our inequity estimates therefore pertain to realized utilization conditional on need and may understate inequity in effective, quality-adjusted care. Relatedly, the survey measures individual-level socioeconomic position; many upstream structural determinants—housing conditions, labor-market security, welfare arrangements—could not be observed directly and are captured only insofar as they operate through income and education.

These limitations point to concrete directions for future research. Establishing the temporal ordering of income, access, and depression requires prospective data; linking future waves of the Türkiye Health Survey or exploiting administrative claims records would permit fixed-effects and cross-lagged panel designs. Most directly, the ongoing expansion of Healthy Life Centers offers a natural opportunity for a stepped-wedge cluster-randomized evaluation of direct physiotherapy access, which would provide causal evidence on whether removing referral barriers reduces the utilization bottleneck documented here—an evaluation design well suited to phased health-system rollouts.

## 5. Policy Implications: Meeting Needs, Creating Opportunities

The evidence presented here speaks directly to the vision of this Special Issue: meeting the needs of diverse communities and creating opportunities for equitable care. While the following recommendations extend beyond what cross-sectional data can directly test, each follows from the specific pattern of barriers identified in the analysis. Türkiye’s health system has created an important opportunity by achieving near-universal coverage; the task now is to translate that structural achievement into people-centered care that provides the most disadvantaged with the services they most need.

Three policy directions emerge from the evidence. First, reducing the socioeconomic gradient in rehabilitation access requires targeting the residual barriers that persist under universal insurance coverage: co-payments and indirect costs for rehabilitation sessions, transportation costs for patients in rural and peri-urban areas [62], the time burden of multiple care episodes, and health literacy gaps that impede navigation of referral pathways. Subsidy programs for transportation and co-payments specifically linked to rehabilitation for low-income households, developed through community engagement with affected populations, would directly address the income-related component of the inequity documented here. For populations with the greatest functional impairment—who face the greatest need but encounter the most severe compounding of socioeconomic barriers—such interventions are not simply equity measures but are essential for realizing the health gains that UHC is intended to produce. Concretely, the Social Security Institution (SGK) could introduce co-payment exemption categories for rehabilitation tied to the means-tested criteria already used in the general health insurance premium support system, while municipal transport authorities could extend existing reduced-fare schemes to documented rehabilitation appointments.

Second, the structural bottleneck between specialist contact and rehabilitation utilization calls for system-level redesign of care pathways [63,64]. Embedding physiotherapists in primary care teams, enabling direct access to physiotherapy for musculoskeletal conditions, and establishing standardized referral protocols from specialist physicians to rehabilitation services would reduce the dependence of rehabilitation access on health literacy and referral navigation—factors that systematically disadvantage lower-educated and lower-income patients. These reforms should be accompanied by workforce development investments to expand the rehabilitation workforce in underserved communities and to ensure that provider availability is not spatially clustered in ways that recreate geographic disadvantages [50]. The Ministry of Health’s Healthy Life Centers [55] provide a ready institutional platform: embedding physiotherapists in these centers with self-referral for uncomplicated musculoskeletal conditions, supported by standardized red-flag screening protocols, would operationalize direct access within the existing public infrastructure.

Third, and perhaps most importantly, the null mediation finding calls for explicit recognition in policy discourse that health system reform, while necessary, is not sufficient to address the mental health burden of poverty. The income–depression gradient in Türkiye operates primarily through direct mechanisms—chronic economic insecurity, material deprivation, and the psychosocial stress of social disadvantage—that are not modified by healthcare access. Social determinants of mental health require social solutions: employment security programs, social housing, income support, food security interventions, and community-based mental health promotion initiatives that address the structural conditions generating depression burden in low-income and marginalized communities. Health systems and social protection systems must be aligned rather than treated as substitutes. Operationally, this argues for joint programming between the Ministry of Health and the Ministry of Family and Social Services—for example, coupling existing conditional cash-transfer and social assistance registries with community mental health outreach—so that households identified through social protection systems are actively connected to preventive mental health support.

Cultural competence in service delivery is also a prerequisite for equitable rehabilitation access. The finding that disadvantages accumulate additively across income and education dimensions suggests that the communities most likely to face barriers include not only the economically poorest but also those with the lowest health literacy, least familiarity with specialist care navigation, and greatest time and transportation constraints [65]. Culturally and linguistically appropriate outreach, care navigation support, and community engagement in the design of rehabilitation services are essential complements to structural reforms in financing and workforce.

## 6. Conclusions

This study makes three empirical contributions. First, it demonstrates that vertical responsiveness and horizontal inequity coexist in Türkiye’s health system: access follows need, yet within every need stratum it also follows income and education, and the decomposition attributes the entirety of the pro-rich gradient to socioeconomic factors, operating additively across the population rather than through isolated intersectional clusters. Second, it identifies and quantifies a structural bottleneck between specialist contact and dedicated rehabilitation: fewer than one in seven of the highest-need individuals reached physiotherapy or rehabilitation services, despite nearly four in five reaching specialist care. Third, it shows that access does not statistically mediate the income–depression gradient, indicating that the mental health burden of poverty operates upstream of healthcare, through chronic economic insecurity and material deprivation.

Together, these findings delineate both the promise and the limits of universal coverage. Formal insurance has secured entry into the system for those with the greatest need, but it has not equalized utilization among equals in need, nor has it converted system entry into need-appropriate rehabilitative care, nor can it, by itself, relieve the mental-health consequences of economic disadvantage. The corresponding policy levers—removing residual financial and navigational barriers, redesigning referral pathways toward direct physiotherapy access, and aligning health policy with social protection—are detailed in Section 5.

Meaningful progress toward people-centered, equitable health systems therefore requires that macroeconomic policy and social protection be recognized as core instruments of health equity, with healthcare reform as a necessary but insufficient complement to the structural transformation of the conditions in which diverse communities live, work, and age.

## Figures and Tables

**Table 1:** Selected characteristics of the analytic sample.

Characteristic	n/Summary	%
Age ≥ 15 (analytic sample)	22,742	100
Female	11,771	51.8
Depression, PHQ-8 ≥ 10	1017	4.5
Disability (Washington Group)	3097	13.6
Broad access (12 months)	14,206	62.5
Narrow access (physiotherapy only)	1263	5.6
Education (highest completed)		
None/low	2369	10.4
Primary–lower-secondary	10,701	47.1
Upper-secondary	5063	22.3
Tertiary	4609	20.3
Household income band (median)	15 of 20	—
Not employed	13,673	60.1

Counts and percentages are unweighted sample descriptives; N = 22,742. Regression, cross-tabulation, concentration index and decomposition estimates reported elsewhere in the manuscript use survey weights. Two exceptions are stated where they arise: the MAIHDA models (Section 2.3, Step 3) and the natural-effect mediation models (Section 2.3, Step 4) are estimated without survey weights.

**Table 2:** Weighted broad-access rate (%) by latent need profile and income tertile.

Need Profile	Low Income (%)	Middle Income (%)	High Income (%)
High Need (Profile 1)	77.3	79.2	81.4
Healthy/Active (Profile 2)	45.3	45.8	53.1
Low Need (Profile 3)	53.3	59.1	63.6

Survey-weighted proportions. Access rises with income within every need profile, demonstrating horizontal inequity.

## Data Availability

Restrictions apply to the availability of these data. Data were obtained from the Turkish Statistical Institute (TurkStat) and are available to researchers upon submission of a formal data access application to TurkStat (https://www.tuik.gov.tr), subject to the terms of the data-use agreement governed by Turkish Statistics Law No. 5429.

## Supplementary Materials

The following supporting information can be downloaded at https://www.mdpi.com/article/10.3390/healthcare14162658/s1. Section S1: data derivation and analytic sample (Table S1); Section S2: confirmatory measurement models (Tables S2 and S3) and PHQ-8 measurement invariance across income, education, and sex groups (Table S4); Section S3: latent profile analysis (Tables S5–S7); Section S4: access models—full coefficients and sensitivity analyses (Tables S8–S12), including NUTS-2 proxy-PSU clustering (Table S10) and classification-uncertainty analyses with proportional assignment and pseudo-class draws (Table S11); Section S5: concentration-index decomposition (Table S13); Section S6: MAIHDA intersectional strata (Table S14); Section S7: functional form—restricted cubic splines and random forest (Tables S15–S17, Figure S1); Section S8: causal mediation—full results (Tables S18–S20, Figure S2); Section S9: statistical software and reproducibility (Table S21).

## Abbreviations

The following abbreviations are used in this manuscript:

AvePP	Average posterior probability
BIC	Bayesian information criterion
CFA	Confirmatory factor analysis
CFI	Comparative fit index
CI	Concentration index
DAG	Directed acyclic graph
EEI	Equal-volume, equal-shape, axis-aligned covariance model
HI	Horizontal-inequity index
ICC	Intraclass correlation coefficient
LPA	Latent profile analysis
LRT	Likelihood-ratio test
MAIHDA	Multilevel analysis of individual heterogeneity and discriminatory accuracy
NDE	Natural direct effect
NIE	Natural indirect effect
NUTS-2	Nomenclature of Territorial Units for Statistics, level 2
OR	Odds ratio
PCV	Proportional change in variance
PHQ-8	Eight-item Patient Health Questionnaire
PSU	Primary sampling unit
RCS	Restricted cubic spline
RMSEA	Root mean square error of approximation
SRMR	Standardized root mean square residual
TLI	Tucker–Lewis index
TurkStat	Turkish Statistical Institute
UHC	Universal Health Coverage
VIF	Variance inflation factor
VPC	Variance partition coefficient
WG	Washington Group short set
WLSMV	Weighted least squares mean- and variance-adjusted estimator
