# Supplementary material for: Social Determinants of Healthcare Access: Horizontal Inequity in Rehabilitation Utilization and the Limits of Care in Mediating the Income–Depression Gradient in Türkiye
Source: Healthcare (Basel). 2026 Aug 21;14(16):2658. doi: 10.3390/healthcare14162658 (PMC13512177; doi:10.3390/healthcare14162658)
Supplement: Supplementary file 1 [file healthcare-14-02658-s001.zip › healthcare-4411844-supplementary.pdf]

Supplementary Materials

Social Determinants of Healthcare Access: Horizontal Inequity in Rehabilitation Utilization and the Limits of Care in Mediating the Income–Depression Gradient in Türkiye

Derya Azim, Muhammed Emre Güvey, Sevde Betül Kara, Sümeyra Gündem, Ecenur Aydemir, Salim Yılmaz

Correspondence: Muhammed Emre Güvey, emre.guvey@acibadem.edu.tr

Overview. This supplementary document provides the extended methodological detail and supporting analyses for the study examining horizontal inequity in rehabilitation and specialist care access in Türkiye and the role of access in mediating the income–depression gradient. Analyses were based on the publicly available micro-data from the 2022 Türkiye Health Survey (TurkStat), restricted to adults aged 15 and over (N = 22,742). The document is organized to follow the analytic chain reported in the main text: data derivation and sample description (Section 1), confirmatory measurement models (Section 2), latent profile analysis (Section 3), access models and sensitivity analyses (Section 4), concentration index decomposition (Section 5), intersectional MAIHDA results (Section 6), functional form analyses (Section 7), causal mediation analyses (Section 8), and statistical software (Section 9).

Abbreviations

Abbreviation	Definition
**AvePP**	Average posterior probability
**AIC**	Akaike information criterion
**BIC**	Bayesian information criterion
**CFA**	Confirmatory factor analysis
**CFI**	Comparative fit index
**CI**	Concentration index (unless stated otherwise; confidence intervals are written out in full)
**CMAverse**	R package for causal mediation analysis
**DAG**	Directed acyclic graph
**EEI**	Equal-volume, equal-shape, axis-aligned covariance model (mclust notation)
**FERTFAKTOR**	Individual sampling weight in the 2022 Türkiye Health Survey
**FMI**	Fraction of missing information
**HI**	Horizontal-inequity index
**ICC**	Intraclass correlation coefficient
**LPA**	Latent profile analysis
**LRT**	Likelihood-ratio test
**MAIHDA**	Multilevel analysis of individual heterogeneity and discriminatory accuracy
**NDE**	Natural direct effect
**NIE**	Natural indirect effect
**NUTS-2**	Nomenclature of Territorial Units for Statistics, level 2 (26 Turkish regions)
**OR**	Odds ratio
**PCV**	Proportional change in variance
**PHQ-8**	Eight-item Patient Health Questionnaire (depression screener)
**PSU**	Primary sampling unit
**RCS**	Restricted cubic spline
**RMSEA**	Root mean square error of approximation
**SRMR**	Standardized root mean square residual
**TLI**	Tucker–Lewis index
**TurkStat**	Turkish Statistical Institute (Türkiye İstatistik Kurumu)
**UHC**	Universal Health Coverage
**VIF**	Variance inflation factor
**VPC**	Variance partition coefficient
**WG**	Washington Group short set on functioning
**WLSMV**	Weighted least-squares mean- and variance-adjusted estimator

Contents

Section S1: Data Derivation and Analytic Sample *(Table S1)*
Section S2: Confirmatory Measurement Models (Tables S2–S4)
Section S3: Latent Profile Analysis *(Tables S5–S7)*
Section S4: Access Models — Full Coefficients and Sensitivity Analyses *(Tables S8–S12)*
Section S5: Concentration-Index Decomposition *(Table S13)*
Section S6: MAIHDA — Intersectional Strata *(Table S14)*
Section S7: Functional Form — RCS and Random Forest *(Tables S15–S17, Figure S1)*
Section S8: Causal Mediation — Full Results *(Tables S18–S20, Figure S2)*
Section S9: Statistical Software and Reproducibility *(Table S21)*
References for Supplementary Material

Section S1: Data Derivation and Analytic Sample

The 2022 Türkiye Health Survey (TurkStat) [15] covers 29,761 household members across all age groups. Applying the age filter (≥15 years) yielded the target subsample of 22,742 adults. Two additional observations were excluded for missing household income, producing an analytic sample of N = 22,740 for multivariable models. Missingness on all other derived analytic variables was negligible (≤0.01%), and complete-case analysis was used throughout. Table S1 documents the derivation counts, raw code distributions for the key constructs, and variable-level missing data rates.

The broad access indicator (REHABILITASYON_ERISIM) was coded as 1 if the respondent reported any visit to a physiotherapist, physical-medicine specialist, or kinesiotherapist, or any specialist-physician contact in the preceding 12 months; 0 otherwise. The narrow (physiotherapy-specific) indicator (REHAB_ERISIM_DAR) was restricted to physiotherapist, physical medicine specialist, and kinesiotherapist contacts only. Depression was operationalized as PHQ-8 ≥ 10 (binary) and as the continuous PHQ-8 sum score (0–24). The five bodily need indicators used in the LPA were: musculoskeletal burden count (KASISKELET_YUK, 0–3); pain score (AGRI_SKOR, 0–5; reverse-coded so higher = more pain); disability-domain count (ENGEL_SAYI, 0–9); self-rated general health (GENEL_SAGLIK_SKOR, 1–5; higher = worse), and a summed weekly activity frequency score across four activity domains (walking, cycling, sport, and muscle strengthening; range 0–28) (FIZIKSEL_AKTIVITE_GUN). Depression (PHQ-8) and both access indicators were excluded from the need construct by design—the Circularity Shield. All population-level estimates were weighted with the individual sampling weight (FERTFAKTOR); the household identifier served as the clustering unit in design-based models because block-level PSU identifiers are withheld from the public-use microdata for confidentiality reasons.

Table S1. Data derivation, raw code distributions, and missing-data rates for key analytic variables.

Variable / Category	n	%	Missing (%)
**SAMPLE DERIVATION**			
Raw data (all ages)	29,761	—	—
Age filter applied (≥15 years)	22,742	100.0	—
Excluded: missing income	2	<0.1	—
Analytic sample (multivariable models)	22,740	99.99	—
**PHQ-8 DEPRESSION SCREENER**			
PHQ-8 total score (range 0–24): median	1.0	—	0.0
PHQ-8 total score: mean (SD)	2.5 (4.0)	—	0.0
Depressive symptoms (PHQ-8 ≥ 10)	1,017	4.5	0.0
No depressive symptoms (PHQ-8 < 10)	21,725	95.5	—

**DISABILITY (WASHINGTON GROUP SHORT SET)**			
Disability present (serious difficulty ≥1 domain)	3,097	13.6	0.0
No disability	19,645	86.4	—
Disability domain count: mean (range 0–9)	0.33 (0–9)	—	0.0
**ACCESS INDICATORS**			
Broad access (specialist or rehabilitation, 12 months)	14,206	62.5	0.0
Narrow access (physiotherapy-specific, 12 months)	1,263	5.6	0.0
— Physiotherapist visits	597	2.6	0.0
— Physical-medicine specialist visits	1,028	4.5	0.0
— Kinesiotherapist visits	191	0.8	0.0
**MUSCULOSKELETAL BURDEN (count 0–3)**			
No condition (score 0)	15,344	67.5	0.0
One condition (score 1)	3,893	17.1	0.0
Two conditions (score 2)	2,782	12.2	0.0
Three conditions (score 3)	723	3.2	0.0
Mean (SD)	0.51 (0.83)	—	—
**BODILY PAIN SCORE (0–5; higher = more pain)**			
Score 0 (no pain)	10,733	47.2	0.0
Score 1	3,760	16.5	0.0
Score 2	2,652	11.7	0.0
Score 3	3,331	14.6	0.0
Score 4	1,685	7.4	0.0
Score 5 (maximum pain)	581	2.6	0.0
Mean (SD)	1.26 (1.48)	—	—
**SOCIOECONOMIC VARIABLES**			
Education: none/low	2,369	10.4	0.0
Education: primary–lower-secondary	10,701	47.1	0.0
Education: upper-secondary	5,063	22.3	0.0
Education: tertiary	4,609	20.3	0.0
Household income band: median (of 20 bands)	15	—	0.0
Not employed	13,673	60.1	0.0
Pain-interference score (AGRI_ENGEL_SKOR)	—	—	47.2

Counts and percentages are unweighted sample descriptives. All regression, cross-tabulation, concentration index, decomposition, and MAIHDA estimates use survey weights (FERTFAKTOR). The pain interference score (AGRI_ENGEL_SKOR) was excluded from all models owing to 47.2% missingness; AGRI_SKOR (severity, 0–5) was used instead. The Circularity Shield specifies that PHQ-8 and both access indicators were excluded from the LPA need construct by design.

Section S2: Confirmatory Measurement Models

Two multi-item constructs were evaluated with confirmatory factor analysis (CFA) using the WLSMV estimator with items treated as ordinal (lavaan [37]). Model fit was assessed against the following thresholds: CFI and TLI ≥ 0.95 (good), ≥ 0.90 (acceptable); RMSEA ≤ 0.06 (good), ≤ 0.08 (acceptable); SRMR ≤ 0.08 (good). The RMSEA is known to be upwardly biased in models with few degrees of freedom estimated on very large samples; it was therefore interpreted alongside the other indices rather than in isolation [19]. Measurement invariance of the PHQ-8 across income, education, and sex groups was additionally tested and is reported in Table S4.

Table S2. PHQ-8 one-factor CFA: global fit indices, internal consistency statistics, and standardized factor loadings (N = 22,742).

Index / Item	Estimate	95% CI / range	Threshold
**GLOBAL FIT INDICES**			
CFI (comparative fit index)	0.980	—	≥ 0.95
TLI (Tucker–Lewis index)	0.973	—	≥ 0.95
RMSEA (scaled)	0.093	[—]	≤ 0.06 a
SRMR	0.041	—	≤ 0.08
χ^2 (scaled)	3,929.8	df = 20, p < 0.001	—
**INTERNAL CONSISTENCY**			
Cronbach α	0.881	—	≥ 0.80
McDonald's ω (total)	0.949	—	≥ 0.80
**STANDARDIZED FACTOR LOADINGS (λ)**			
Anhedonia (ZEVK_DUYMA)	0.849	p < 0.001	—
Depressed mood (BUNALIM)	0.887	p < 0.001	—
Sleep problems (UYKU_SORUN)	0.799	p < 0.001	—
Fatigue (YORGUNLUK)	0.846	p < 0.001	—
Appetite disturbance (ISTAHSIZLIK)	0.800	p < 0.001	—
Worthlessness (DEGERSIZ_HISSETME)	0.890	p < 0.001	—
Concentration problems (KONSANTRASYON_PRB)	0.852	p < 0.001	—
Psychomotor disturbance (HUZURSUZLUK)	0.831	p < 0.001	—
Item-total correlations (range)	0.555–0.727	—	—

WLSMV estimator; items treated as ordinal. a—The elevated RMSEA (0.093) reflects the known upward bias of this index at low degrees of freedom (df = 20) in very large samples [19]. Given that CFI, TLI, and SRMR all indicate good fit, the PHQ-8 was retained as a summed score. The binary depression indicator uses the conventional PHQ-8 threshold of ≥ 10 for moderate-or-greater depressive symptoms [16].

Table S3. Washington Group (WG) short set one-factor CFA: global fit indices and standardized factor loadings, and justification for operationalizing disability as a domain-count index (N = 22,690 complete).

Index / Item	Estimate	95% CI / range	Threshold
**GLOBAL FIT INDICES**			
CFI	0.980	—	≥ 0.95
TLI	0.973	—	≥ 0.95
RMSEA (scaled)	0.123	—	≤ 0.06
SRMR	0.166	—	≤ 0.08
χ^2 (scaled)	9,312.9	df = 27, p < 0.001	—
**INTERNAL CONSISTENCY**			
Cronbach α	0.850	—	≥ 0.80
McDonald's ω (total)	0.940	—	≥ 0.80
**STANDARDIZED FACTOR LOADINGS (λ) — note heterogeneity**			
Vision (GORMEDE_ZORLUK)	0.542	p < 0.001	—
Hearing in silence (DUYMA_SESSIZLIK)	0.984	p < 0.001	—
Hearing in noise (DUYMA_GURULTU)	0.934	p < 0.001	—
Walking 500 m without support	0.945	p < 0.001	—
Climbing 12 steps without support	0.972	p < 0.001	—
Carrying/holding objects	0.913	p < 0.001	—
Remembering (HATIRLAMA)	0.713	p < 0.001	—
Speaking (KONUSMA)	0.889	p < 0.001	—
Communication in native language	0.915	p < 0.001	—
Item-total correlations (range)	0.400–0.753	—	—

WLSMV estimator. The WG set is explicitly designed to measure distinct functioning domains rather than a single latent trait; heterogeneous loadings (vision λ = 0.54 vs. hearing λ = 0.98) and poor absolute fit (SRMR = 0.166, RMSEA = 0.123) are consistent with this design intent and are not indicators of item problems per se. The WG instrument is therefore operationalized as a domain-count index (ENGEL_SAYI, range 0–9) and a binary disability indicator (serious difficulty in ≥ 1 domain), following standard WG methodology [17], rather than as a latent factor score.

To verify that PHQ-8 summed scores are comparable across the socioeconomic groups central to the analysis, measurement invariance was tested with multi-group ordinal CFA under the Wu–Estabrook [20] identification (configural → thresholds → thresholds + loadings), with decisions based on ΔCFI ≥ −0.010 and ΔRMSEA ≤ +0.015 [21,22] rather than on scaled χ^2 difference tests, which are oversensitive at N = 22,742; the χ^2 value and degrees of freedom of every model are reported in Table S4.

Table S4. Measurement invariance of the PHQ-8 across income tertiles, education groups, and sex: multi-group ordinal CFA (WLSMV, delta parameterization, and Wu–Estabrook identification).

Grouping	Model	χ^2 (df)	CFI	TLI	RMSEA	SRMR	ΔCFI	ΔRMSEA	Decision
Income tertile (3 groups)	M1 Configural	3916.0 (60)	0.981	0.973	0.092	0.043	—	—	—
	M2 Thresholds	4211.5 (76)	0.979	0.977	0.085	0.043	−0.0014	−0.0074	Supported
	M3 Thresholds + loadings	3329.0 (90)	0.984	0.985	0.069	0.043	+0.0045	−0.0158	Supported
Education (4 levels) [a]	M1 Configural	3732.4 (80)	0.982	0.974	0.090	0.045	—	—	—
	M2 Thresholds [a]	3732.4 (80)	0.982	0.974	0.090	0.045	0.0000	0.0000	Supported
	M3 Thresholds + loadings	3569.8 (101)	0.983	0.981	0.078	0.045	+0.0009	−0.0119	Supported
Sex (2 groups)	M1 Configural	3936.3 (40)	0.980	0.972	0.093	0.042	—	—	—
	M2 Thresholds	4147.9 (48)	0.979	0.975	0.087	0.042	−0.0011	−0.0059	Supported
	M3 Thresholds + loadings	3454.5 (55)	0.982	0.982	0.074	0.042	+0.0037	−0.0130	Supported

a—For the education grouping, response categories 2 and 3 were collapsed (0–1–2 scale) owing to a sparse cell (n = 9 in the highest response category of one item among tertiary-educated respondents); with three response categories, the threshold-constrained model is statistically equivalent to the configural model under the Wu–Estabrook identification, yielding identical fit. Δ values were computed from unrounded fit indices and are reported to four decimal places; they may therefore differ slightly from differences calculated using the three-decimal values shown. "Supported" = ΔCFI ≥ −0.010 and ΔRMSEA ≤ +0.015 relative to the preceding model. Full threshold-and-loading invariance was supported for all three groupings; PHQ-8 scores are therefore comparable across income, education, and sex groups.

Section S3: Latent Profile Analysis

Latent profile analysis (LPA) was estimated on the five bodily need indicators using Gaussian finite-mixture models (mclust package, R). Four covariance parameterizations were evaluated: EEI (equal variance, axis-aligned; primary), VVI (varying variance, axis-aligned), EEE (equal full covariance), and VVV (varying full covariance). Models with one to six profiles were fitted for each parameterization where estimable. VVI and VVV with two or more profiles were non-estimable owing to the zero-inflated count indicators. EEE and VVV with three or more profiles produced empty classes. Selection of the three-profile EEI solution was based on four criteria considered jointly: the BIC elbow, entropy, smallest-class size, and substantive interpretability. Table S5 presents the full fit statistics across all estimable models.

Table S5. Latent profile analysis model comparison: log-likelihood, AIC, BIC, entropy, minimum class size (n), and minimum classification probability for one-to-six profiles under four covariance parameterizations.

Model	Profiles	LogLik	AIC	BIC	Entropy	n (min)	Prob (min)

EEI	1	-161,345.0	322,710.0	322,790.3	1.000	22,742	1.000
EEI	2	-148,370.3	296,772.5	296,901.0	0.921	4,679	0.505
**EEI**	**3 ***	**-136,439.0**	**272,922.0**	**273,098.7**	**0.680**	**1,665**	—
EEI	4	-136,172.8	272,401.7	272,626.6	0.751	2,245	0.500
EEI	5	-132,737.6	265,543.2	265,816.3	0.985	327	0.500
EEI	6	-133,919.7	267,919.4	268,240.6	0.683	606	0.295
VVI	1	-161,345.0	322,710.0	322,790.3	1.000	22,742	1.000
VVI	2–6	Non-estimable (zero-inflated indicators)					
EEE	1	-150,851.1	301,742.1	301,902.8	1.000	22,742	1.000
EEE	2	-150,051.5	300,155.0	300,363.8	0.960	661	0.500
EEE	3	-150,050.7	300,165.5	300,422.5	0.823	0	0.496
EEE	4–6	Empty class(es) produced					
VVV	1	-150,851.1	301,742.1	301,902.8	1.000	22,742	1.000
VVV	2–6	Non-estimable (zero-inflated indicators)					

**Selected model. BIC elbow occurs between two and three profiles in the EEI family. The three-profile EEI solution was preferred over the five-profile EEI (lowest BIC) on grounds of interpretability and the very small minimum class size (n = 327, 1.4%) in the five-profile solution. VVI and VVV models with ≥ 2 profiles did not converge due to zero-inflated count indicators. EEE and VVV models with ≥ 3 profiles produced degenerate (empty) classes. The equal-covariance EEE family was dominated by the EEI family on BIC at every profile count. Entropy for the selected three-profile EEI solution = 0.68 (see Table S6 for classification quality details).*

Table S6. Average posterior probability (AvePP) matrix and posterior-probability distributions for the selected three-profile EEI solution. Rows = modal (assigned) class; columns = mean posterior probability for each profile.

Modal class	Post. P1 (high need)	Post. P2 (healthy/active)	Post. P3 (low need)
Profile 1 — High need (n = 4,406)	0.968 *	0.001	0.031
Profile 2 — Healthy/active (n = 1,665)	0.001	0.688 △	0.311
Profile 3 — Low need (n = 16,671)	0.023	0.146	0.832 *

**AvePP ≥ 0.80: acceptable classification quality. △ AvePP = 0.69, below the 0.80 threshold; overlap is confined to the boundary between the two low-need profiles (P2 and P3). Profile-level posterior-probability distributions: Profile 1 — median max-posterior = 1.000 (Q1 0.994; only 1.2% of assigned cases with max-posterior < 0.60); Profile 2 — median 0.647 (Q1 0.551; 45.7% of cases with max-posterior < 0.60); Profile 3 — median 0.858 (Q1 0.737; 5.6% < 0.60). The low classification quality in Profile 2 reflects overlap with Profile 3, both representing low bodily burden. Because Profile 1 — the high-need profile that anchors the main equity findings — shows near-perfect classification (AvePP = 0.968), and because a proportional-assignment sensitivity analysis (Table S11) yielded an identical income OR (1.021), the moderate overall entropy does not bias the primary conclusions.*

Table S7. Raw and standardized (z-score) indicator means by latent need profile (EEI three-profile solution, N = 22,742).

Indicator	P1 High need (n=4,406)	P2 Healthy/ active (n=1,665)	P3 Low need (n=16,671)	Overall mean	Overall SD	z P1	z P2

Musculoskeletal burden (0–3)	1.95	0.06	0.18	0.51	0.83	+1.74	−0.54
Pain score (0–5)	2.86	0.30	0.94	1.26	1.48	+1.08	−0.65
Disability-domain count (0–9)	1.19	0.02	0.13	0.33	1.04	+0.83	−0.29
Self-rated health (1–5; higher=worse)	3.13	1.54	2.27	2.39	0.77	+0.97	−1.10
Activity-frequency score (0–28)	3.81	11.13	4.34	4.73	3.58	−0.26	+1.79

Standardized z-scores computed relative to the overall sample mean and SD. Profile 1 (High Need) is characterized by substantially elevated musculoskeletal burden, pain, disability, and poor self-rated health, and by below-average physical activity. Profile 2 (Healthy/Active) shows the lowest burden across all five indicators and the highest activity frequency score (mean 11.1 on a 0–28 scale summing days per week across four separate activity domains). Profile 3 (Low Need/General Population) approximates the overall sample mean across all indicators. These profiles served as the parallel categorical operationalization of bodily need in the access models (Model B). The activity frequency score sums the number of days per week reported separately for four domains — walking ≥10 minutes, cycling, sport, and muscle-strengthening activities — and therefore ranges from 0 to 28 rather than 0 to 7.

Section S4: Access Models — Full Coefficients and Sensitivity Analyses

Four logistic models were estimated for broad access. Model 0 (socioeconomic only) included income, education, and demographic covariates without need adjustment. Model A (primary) added continuous need indicators (musculoskeletal burden, pain score, disability-domain count, self-rated health). Model B (parallel) replaced continuous need indicators with the three latent need profiles. A narrow-access sensitivity model used the Model A specification applied to physiotherapy-specific access. All models used survey-weighted logistic regression (svyglm, quasibinomial family) with household-level clustering and FERTFAKTOR weights. Tables S8 and S9 present full coefficients; Tables S10 and S12 report sensitivity analyses. Classification-uncertainty sensitivity analyses (proportional assignment and pseudo-class draws) are reported in Table S11.

Table S8. Complete odds ratios (OR) with 95% confidence intervals and p-values for Models 0, A, and B (broad access; survey-weighted, household-clustered; N = 22,740).

Predictor	M0 OR	M0 95% CI	M0 p	MA OR	MA 95% CI	MA p	MB OR	MB 95% CI	MB p
**SOCIOECONOMIC**									
Income (per band)	1.017	1.009–1.025	<0.001	1.027	1.018–1.035	<0.001	1.021	1.013–1.029	<0.001
Education: none/low (ref: tertiary)	0.698	0.601–0.811	<0.001	0.481	0.413–0.560	<0.001	0.598	0.514–0.695	<0.001
Education: primary–lower-secondary	0.737	0.672–0.808	<0.001	0.646	0.588–0.709	<0.001	0.696	0.635–0.763	<0.001
Education: upper-secondary	0.885	0.804–0.974	0.012	0.832	0.755–0.917	<0.001	0.860	0.782–0.947	0.002
**NEED INDICATORS**									
Musculoskeletal burden	—	—	—	1.186	1.128–1.246	<0.001	—	—	—
Pain score	—	—	—	1.223	1.189–1.257	<0.001	—	—	—
Disability-domain count	—	—	—	1.019	0.977–1.064	0.374	—	—	—
Self-rated health (worse = higher)	—	—	—	1.651	1.562–1.746	<0.001	—	—	—
Need profile 1 (high need; ref: P3)	—	—	—	—	—	—	2.441	2.221–2.682	<0.001

Need profile 2 (healthy/active)	—	—	—	—	—	—	0.732	0.651–0.823	<0.001
**DEMOGRAPHIC COVARIATES**									
Female (ref: male)	1.537	1.444–1.637	<0.001	1.321	1.238–1.410	<0.001	1.396	1.310–1.487	<0.001
Age (per year)	1.008	1.006–1.011	<0.001	0.995	0.993–0.998	0.002	1.004	1.001–1.006	0.011
Marital: never married (ref: married)	0.711	0.647–0.781	<0.001	0.724	0.658–0.797	<0.001	0.729	0.663–0.802	<0.001
Marital: divorced	1.014	0.868–1.185	0.858	1.023	0.872–1.200	0.780	1.016	0.869–1.188	0.843
Marital: widowed	1.159	0.998–1.347	0.054	1.062	0.905–1.246	0.464	1.111	0.952–1.296	0.182
Not employed (ref: employed)	1.096	1.020–1.179	0.013	1.060	0.984–1.143	0.123	1.093	1.016–1.175	0.017
No social-security cover (ref: covered)	0.698	0.613–0.795	<0.001	0.681	0.596–0.778	<0.001	0.692	0.606–0.789	<0.001
**MODEL FIT**									
McFadden pseudo-R^2 (unweighted)	0.026	—	—	0.074	—	—	0.044	—	—
LRT vs. Model 0	—	—	—	$\Delta\chi^2$=1,423.4	df=4	p<0.001	—	—	—

All VIF < 2.3 (no multicollinearity concern). Survey-weighted estimates using FERTFAKTOR; household identifier as clustering unit. LRT = likelihood-ratio test (unweighted models). M0 = Model 0 (socioeconomic only); MA = Model A (continuous need; primary); MB = Model B (latent profile). Reference categories: education = tertiary; sex = male; marital status = currently married; employment = employed; social-security = covered. "—" = predictor not included in that model.

Table S9. Variance inflation factors (VIF) for Model A (continuous need; primary specification).

Predictor	GVIF	GVIF^(1/(2×df))
Income (per band)	1.30	1.14
Education (3 df)	1.62	1.08
Musculoskeletal burden	1.35	1.16
Pain score	1.38	1.17
Disability-domain count	1.33	1.15
Self-rated health	1.53	1.24
Sex	1.28	1.13
Age	2.29	1.51
Marital status (3 df)	2.18	1.14
Employment status	1.41	1.19
Social-security coverage	1.05	1.02

All GVIF^(1/(2×df)) values well below 2.0, confirming the absence of multicollinearity. GVIF (generalized VIF) is used for categorical predictors with more than one degree of freedom; standard VIF is used for continuous and binary predictors.

Table S10. Sensitivity analysis for the primary income odds ratio: NUTS-2 proxy-PSU clustering.

Predictor	OR	95% CI	p	Notes
**NUTS-2 proxy-PSU sensitivity (Model A; 26 NUTS-2 regions as clustering unit)**				

Income (per band)	1.027	1.012–1.042	0.002	CI width ratio NUTS-2/household = 1.76
Education: none/low	0.481	0.390–0.593	<0.001	All conclusions preserved
Education: primary–lower-secondary	0.646	0.576–0.725	<0.001	—
Education: upper-secondary	0.832	0.702–0.986	0.037	—
Musculoskeletal burden	1.186	1.068–1.316	0.005	—
Pain score	1.223	1.186–1.261	<0.001	—
Disability-domain count	1.019	0.965–1.077	0.451	—
Self-rated health (worse)	1.651	1.513–1.802	<0.001	—
Female (ref: male)	1.321	1.234–1.414	<0.001	—
No social-security cover	0.681	0.604–0.768	<0.001	—

NUTS-2 regions (n = 26) used as proxy PSU in place of the unreleased block-level identifiers. Household-level ICC = 29.4%; NUTS-2-level ICC = 1.8%, confirming the household as the dominant clustering unit. The income OR point estimate is unchanged (1.027); CI widening (ratio 1.76) reflects the additional between-region variance absorbed at NUTS-2 level.

Table S11. Classification-uncertainty sensitivity analyses for the profile-adjusted access model (Model B): (a) income coefficient across three membership-assignment methods; (b) pooled pseudo-class estimates, full model.

Method	Income OR (per band)	95% CI	FMI (%)
Modal assignment (main Model B)	1.021	1.013–1.029	—
Proportional assignment (posterior-weighted) b	1.021	1.016–1.025	—
Pseudo-class draws (K = 50, Rubin's rules)	1.021	1.012–1.029	0.30

Panel (b). Pooled pseudo-class estimates (Rubin's rules; reference categories: low-need profile, tertiary education, male, married, employed, covered).

Term	OR	95% CI	FMI (%)
Income (per band)	1.021	1.012–1.029	0.30
Education: none/low	0.601	0.517–0.699	0.38
Education: primary–lower-secondary	0.702	0.640–0.769	0.36
Education: upper-secondary	0.865	0.786–0.953	0.28
High-need profile (vs. low-need)	2.399	2.175–2.645	10.77
Healthy/active profile (vs. low-need)	0.848	0.758–0.949	45.50
Female (vs. male)	1.407	1.320–1.500	1.01
Age (per year)	1.004	1.001–1.006	1.02
Marital status: never married	0.725	0.659–0.797	0.31
Marital status: divorced	1.009	0.862–1.181	0.29
Marital status: widowed	1.103	0.945–1.288	0.46
Not employed	1.089	1.012–1.172	0.26
No social-security coverage	0.690	0.605–0.787	0.18

b Expanded-data confidence interval shown for point-estimate comparison; it does not incorporate the survey design. FMI = fraction of missing information attributable to classification uncertainty, from Rubin's rules pooling across K = 50 pseudo-class draws [26,27]; each draw re-estimated the survey-weighted, household-clustered model with profile membership sampled from the individual posterior distribution (fixed seed 20260709). FMI is concentrated in the contrast between the two low-burden profiles, consistent with the AvePP pattern in Table S6 (healthy/active AvePP = 0.69), whereas the high-need profile (AvePP = 0.97) and all socioeconomic coefficients are essentially unaffected.

Table S12. Narrow-access sensitivity model (physiotherapy-specific access; Model A specification; N = 22,740; survey-weighted, household-clustered).

Predictor	OR	95% CI	p	Notes
Income (per band)	1.027	1.012–1.042	0.001	Income gradient preserved under narrow definition
Education: none/low (ref: tertiary)	0.538	0.406–0.714	<0.001	Steeper education gradient than broad access
Education: primary–lower-secondary	0.646	0.536–0.777	<0.001	—
Education: upper-secondary	0.823	0.677–1.002	0.052	Borderline
Musculoskeletal burden	1.707	1.588–1.835	<0.001	Stronger need effect (condition-specific pathway)
Pain score	1.296	1.231–1.365	<0.001	—
Disability-domain count	1.008	0.953–1.066	0.776	Non-significant
Self-rated health (worse)	1.174	1.056–1.306	0.003	Attenuated vs. broad access
Female (ref: male)	1.023	0.882–1.187	0.761	Female excess disappears (vs. OR 1.321 in broad)
Age (per year)	0.999	0.994–1.005	0.833	Non-significant
Marital: never married	0.854	0.684–1.066	0.164	—
Marital: divorced	1.526	1.159–2.009	0.003	Significant (specific to narrow)
Marital: widowed	0.875	0.668–1.146	0.332	—
Not employed	1.255	1.069–1.473	0.006	—
No social-security cover	0.862	0.655–1.136	0.293	Non-significant (narrow access)

Narrow access prevalence = 5.6% (N = 1,263). The disappearance of the female excess (OR 1.023, ns) under the narrow definition, contrasted with the broad-access female excess (OR 1.321), confirms that the broad-access female advantage reflects greater specialist-physician utilization rather than physiotherapy-specific access. The stronger musculoskeletal effect (OR 1.707 vs. 1.186) confirms that the narrow definition better captures the condition-specific rehabilitation pathway. All other directions preserved.

Section S5: Concentration Index Decomposition

The Erreygers-corrected concentration index (E_C) was the primary measure of income-related inequality in access, with the standard concentration index (CI) reported as a secondary measure. Both were computed analytically following standard methodology [2,3] and cross-validated with the rineq package [39]. Because the Erreygers correction rescales the standard index by a constant ($4\mu \times$ CI for a binary outcome), percentage contributions are identical under both indices, ensuring that the Wagstaff decomposition is invariant to the choice between them. Table S13 presents the full determinant-level decomposition.

Table S13. Full Wagstaff decomposition of the concentration index for broad access by individual determinant. Primary index: Erreygers-corrected E_C = 0.058 (μ = 0.616; 4μ = 2.463); secondary: standard CI = 0.023. Cross-validation with the rineq package reproduced both indices exactly (type 'CI' = 0.0234; type 'CIc' = 0.0577).

Variable	β	$\bar{x}$	CI_k	Elasticity	Contrib. (raw)	Contrib. (Err.)	% of CI
**SOCIOECONOMIC (pro-rich contribution)**							
Income (GELIR_GRUP_SAYI)	0.0065	14.370	+0.2015	0.1524	+0.0307	+0.0756	+131.2%
Education (EGITIM_DUZEY)	0.0483	2.512	+0.0864	0.1972	+0.0170	+0.0419	+72.8%

**Socioeconomic subtotal**	—	—	—	—	**+0.0477**	**+0.1175**	**+204.0%**
**NEED (pro-poor contribution — offsets pro-rich gradient)**							
Self-rated health (GENEL_SAGLIK_SKOR)	0.1047	2.373	−0.0340	0.4035	−0.0137	−0.0338	−58.5%
Pain score (AGRI_SKOR)	0.0418	1.240	−0.0839	0.0842	−0.0071	−0.0174	−30.2%
Musculoskeletal burden (KASISKELET_YUK)	0.0329	0.498	−0.0976	0.0266	−0.0026	−0.0064	−11.1%
Disability-domain count (ENGEL_SAYI)	−0.0049	0.328	−0.2801	−0.0026	+0.0007	+0.0018	+3.1%
**Need subtotal**	—	—	—	—	**−0.0227**	**−0.0558**	**−96.7%**
**DEMOGRAPHIC**							
Sex (CINSIYET_F)	0.0722	0.505	−0.0253	0.0592	−0.0015	−0.0037	−6.4%
Age (YAS)	0.0005	42.444	−0.0341	0.0352	−0.0012	−0.0030	−5.1%
**Demographic subtotal**	—	—	—	—	**−0.0027**	**−0.0067**	**−11.5%**
**TOTAL (explained + residual)**							
**Explained component**	—	—	—	—	**+0.0224**	**+0.0552**	**96.0%**
**Residual**	—	—	—	—	**+0.0010**	**+0.0025**	**4.0%**

β: regression coefficient for broad access (svyglm); $\bar{x}$: weighted mean; CI_k: own concentration index of each determinant (positive = pro-rich distribution, negative = pro-poor distribution); Elasticity = β × $\bar{x}$ / μ where μ = 0.616 is the weighted access prevalence; Contrib. (raw) = Elasticity × CI_k; Contrib. (Err.) = Contrib. (raw) × 4μ = Contrib. (raw) × 2.463. Percentage contributions are identical under raw and Erreygers indices (scaling invariance). The horizontal-inequity index (HI) equals the socioeconomic subtotal contribution: Erreygers HI = +0.1175; raw HI = +0.0477 (direct need-standardization: raw HI = +0.044). HI > E_C indicates that need is pro-poor and offsets the pro-rich gradient — standardizing for need amplifies measured inequity.

Section S6: MAIHDA — Intersectional Strata

Multilevel analysis of individual heterogeneity and discriminatory accuracy (MAIHDA) was applied to examine whether inequalities in access are generated by broadly acting socioeconomic gradients or concentrated in specific intersectional subgroups. Individuals were assigned to 36 strata defined by sex (2) × income tertile (3) × age group (3: young/mid/old) × education group (2: low/high, collapsing the four-category variable into ≤primary–secondary vs. ≥upper-secondary/tertiary). Two-level logistic models were estimated without survey weights, consistent with standard MAIHDA practice [30–32]. The null model estimated between-stratum variance only; the main-effects model added all socioeconomic, demographic, and need covariates. The variance partition coefficient (VPC) quantifies the proportion of individual variation in access explained by stratum membership; the proportional change in variance (PCV) captures how much of that between-stratum variance is explained by the main effects. Table S14 presents predicted access for all 36 strata.

Interpreting the MAIHDA results. The null-model VPC of 3.8% indicates that only a small fraction of the variation in access lies between intersectional strata — most variation is within-stratum individual heterogeneity. The PCV of 97.2% after adding main effects means that virtually all between-stratum variance is attributable to the additive effects of income, education, age, and sex individually, leaving a residual pure-interaction component of only 2.8% of the original between-stratum variance. This is the defining feature of an additive, gradient-driven inequality pattern: disadvantage accumulates through the main effects of each social position rather than through unique, emergent penalties at specific intersections. The strata ranked lowest on predicted access (young, low-income, low-education men) and highest (older, higher-income, higher-education women) reflect the main-effect ordering rather than unexpected intersectional concentrations.

Table S14. MAIHDA null-model predicted broad-access probability for all 36 intersectional strata, ordered from lowest to highest, with stratum size (n). VPC (null model) = 3.8%; PCV after main effects = 97.2%; implied pure interaction = 2.8%.

Stratum (sex · income · age · education)	Predicted access (probability)	n
Male · low income · young · low education	0.447	679
Male · mid income · young · low education	0.483	573
Male · low income · young · high education	0.495	520
Male · low income · mid age · low education	0.515	895
Male · low income · mid age · high education	0.516	329
Male · high income · young · low education	0.528	294
Male · mid income · mid age · low education	0.539	863
Male · mid income · young · high education	0.543	895
Male · mid income · mid age · high education	0.566	727
Female · mid income · young · low education	0.580	592
Female · low income · young · low education	0.583	762
Male · high income · young · high education	0.600	940
Female · high income · young · low education	0.605	246
Male · high income · mid age · low education	0.606	325
Female · low income · young · high education	0.608	569
Male · high income · mid age · high education	0.616	966
Male · low income · old age · low education	0.619	1,175
Male · mid income · old age · low education	0.632	732
Male · mid income · old age · high education	0.634	304
Female · low income · old age · high education	0.637	75
Female · low income · mid age · low education	0.644	1,179
Male · low income · old age · high education	0.657	128
Male · high income · old age · low education	0.670	280
Female · mid income · mid age · low education	0.678	1,099
Female · mid income · young · high education	0.678	916
Female · high income · young · high education	0.689	915
Female · high income · mid age · low education	0.697	496
Female · low income · old age · low education	0.701	1,632
Female · mid income · old age · high education	0.705	168
Female · mid income · mid age · high education	0.707	568
Male · high income · old age · high education	0.709	346
Female · high income · old age · low education	0.718	352
Female · low income · mid age · high education	0.720	233
Female · high income · mid age · high education	0.730	849
Female · mid income · old age · low education	0.737	894
Female · high income · old age · high education	0.760	224

Unweighted two-level logistic models (lme4). Strata with the lowest predicted access (shaded amber, ranks 1–8) are consistently young, low-income, low-education men. Strata with the highest predicted access (shaded green, ranks 29–36) are consistently older, higher-income, higher-education women. The gradient across strata is gradual and monotone, reflecting additive socioeconomic effects rather than non-linear intersectional concentrations. No stratum size fell below n = 75 (minimum cell: Female · low income · old age · high education). Income tertile cut-points: low = bands 1–7 (n ≈ 7,600), mid = bands 8–14, high = bands 15–20. Age groups: young ≤ 34, mid 35–54, old ≥ 55. Education: low = none/low + primary–lower-secondary; high = upper-secondary + tertiary.

Section S7: Functional Form — Restricted Cubic Splines and Random Forest

To assess whether the linear income specification adequately characterizes the income–access relationship, two complementary approaches were used: (a) restricted cubic splines (RCS) within the survey-weighted logistic framework as the primary parametric test, and (b) a probability random forest with partial-dependence analysis as a non-parametric supplementary check. The two approaches converge on the same conclusion: the income–access relationship is predominantly linear, with a modest plateau at lower income levels.

Table S15. Restricted cubic spline (RCS) linearity tests for the income–access relationship within the survey-weighted logistic model (Model A specification; household clustering; N = 22,740).

Test	Statistic	df (num / den)	p-value	Interpretation
RCS df=3: Wald test on non-linear terms (survey-weighted)	F = 1.40	2 / 10,009	p = 0.246	No evidence of departure from linearity
RCS df=4: Wald test on non-linear terms (survey-weighted)	F = 1.25	3 / 10,008	p = 0.290	Confirmed in more flexible specification
LRT: Linear vs. Spline(3) (unweighted)	$\Delta\chi^2$ = 8.48	df = 2	p = 0.014	Marginal improvement; does not account for survey design
LRT: Linear vs. Spline(4) (unweighted)	$\Delta\chi^2$ = 10.53	df = 3	p = 0.015	Consistent with df=3 result
PDP linear-fit R^2 (random forest)	R^2 = 0.919	—	—	Near-linear partial-dependence profile

The survey-weighted Wald tests evaluate only the non-linear basis terms (2 and 3 degrees of freedom respectively) and therefore test departure from linearity directly; neither reaches significance. The unweighted likelihood-ratio tests, which do not account for the complex survey design, indicate a marginal improvement in fit. Predicted access curves from the linear and spline specifications overlapped closely (Figure S1): the spline reveals a flat region across lower income bands (≈ bands 1–10) followed by a steady rise above the median, consistent with the random-forest partial-dependence profile (Table S16). The linear specification was therefore retained, supported by both the design-based tests and the PDP R^2 of 0.92 indicating that a straight line captures 92% of the income-gradient shape.

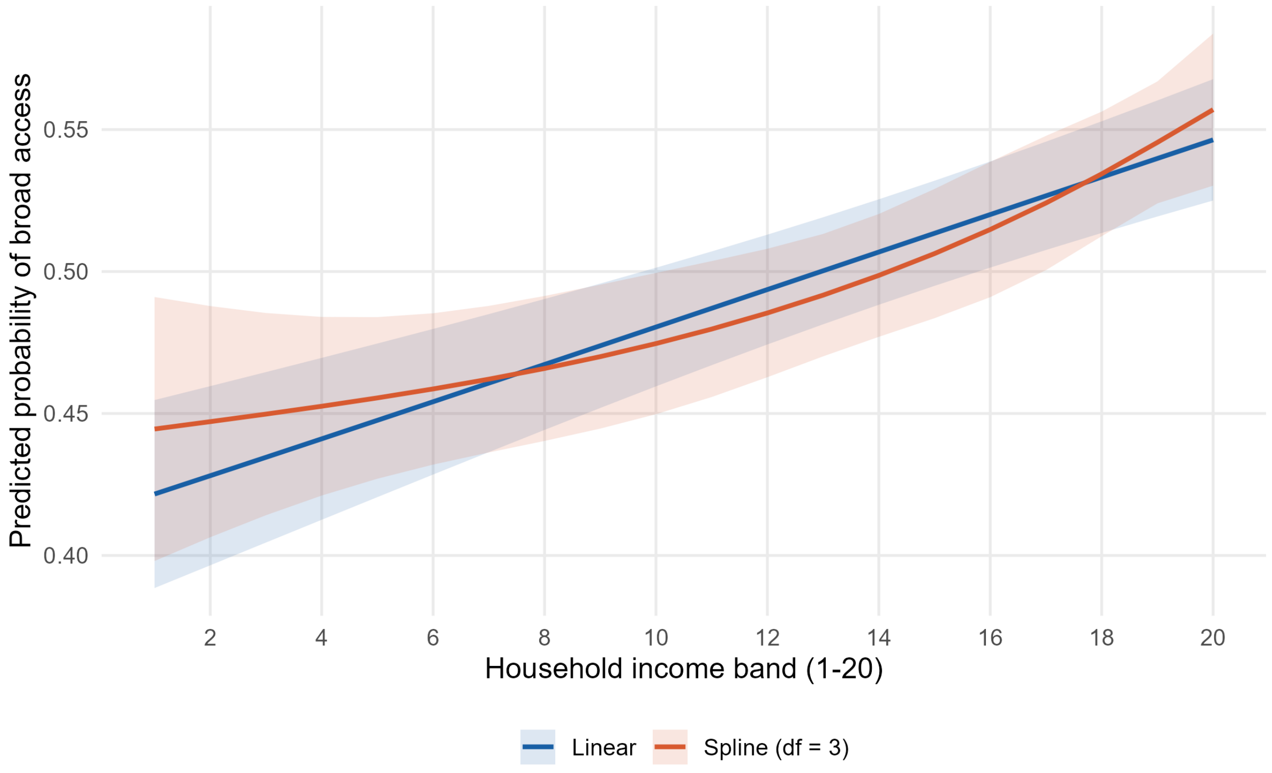

Figure S1. Predicted probability of broad access across household income bands under the linear and the restricted cubic spline (df = 3) specifications of the survey-weighted logistic model. Shaded regions denote 95% confidence intervals.

Table S16. Random-forest partial-dependence values: predicted probability of broad access at each income band (1–20), with all other predictors held at their observed values. OOB Brier score = 0.214.

Income band	Predicted access (PDP)	Slope (Δ predicted access from previous band)

1 (lowest)	0.547	— (reference)
2	0.570	+0.023 (steepest rise in lower half)
3	0.567	−0.003
4	0.557	−0.010
5	0.556	−0.002
6	0.556	+0.001
7	0.576	+0.020
8	0.566	−0.010
9	0.568	+0.002
10	0.585	+0.017
11	0.601	+0.015 (gradient steepens above median)
12	0.604	+0.003
13	0.607	+0.004
14	0.613	+0.006
15	0.625	+0.012
16	0.628	+0.003
17	0.629	+0.001
18	0.640	+0.010
19	0.649	+0.009
20 (highest)	0.659	+0.010

Probability random forest (ranger; 500 trees; permutation variable importance). The partial-dependence profile shows an approximately flat region across income bands 1–10 (plateau: predicted access ≈ 0.55–0.59) followed by a roughly linear rise from band 10 to 20 (0.59 → 0.66). This mild plateau-then-rise pattern is consistent with the absence of significant departure from linearity in the design-based RCS tests (Table S15) and supports the interpretation that the income gradient is approximately linear above the median income, while the lowest-income households face a floor effect.

Table S17. Random-forest permutation variable importance for predicting broad access (higher = more important).

Variable	Permutation importance	Category
Self-rated health (GENEL_SAGLIK_SKOR)	0.01870	Need
Pain score (AGRI_SKOR)	0.01129	Need
Musculoskeletal burden (KASISKELET_YUK)	0.00891	Need
Age (YAS)	0.00659	Demographic
Education (EGITIM_DUZEY)	0.00553	Socioeconomic
Income (GELIR_GRUP_SAYI)	0.00512	Socioeconomic
Sex (CINSIYET_F)	0.00476	Demographic
Disability count (ENGEL_SAYI)	0.00428	Need
Employment (CALISMA_F)	0.00320	Socioeconomic
Social-security coverage (SGK_VAR)	0.00163	Socioeconomic

Need indicators (self-rated health, pain, musculoskeletal burden) dominate variable importance, confirming the system is primarily responsive to clinical need. Among socioeconomic factors, education ranks slightly above income in permutation importance, consistent with the decomposition finding that education contributes approximately 73% and income 131% to the concentration index (the relative importance difference reflects the differing scales of the two variables). The random-forest analysis is presented as a distributional-assumption-free supplementary check; primary inference uses the survey-weighted logistic models.

Section S8: Causal Mediation — Full Results

The income → access → depression mediation pathway was examined with two complementary estimators: natural-effect models (medflex package) and a regression-based approach with exposure–mediator interaction (CMAverse package). Both estimators adjusted for the same set of baseline confounders: education, musculoskeletal burden, pain score, disability-domain count, self-rated health, sex, age, and employment status. The assumed directional structure is formalized in a directed acyclic graph (Figure S2): income and education are treated as temporally prior to access and depression; bodily need is modeled as a confounder of the access–depression relationship; the access–depression arrow is marked as potentially bidirectional to acknowledge possible confounding by indication inherent in the cross-sectional design. All estimates are for a per-band increment in income (exposure x = 1, x* = 0). Tables S18–S20 present the full results.

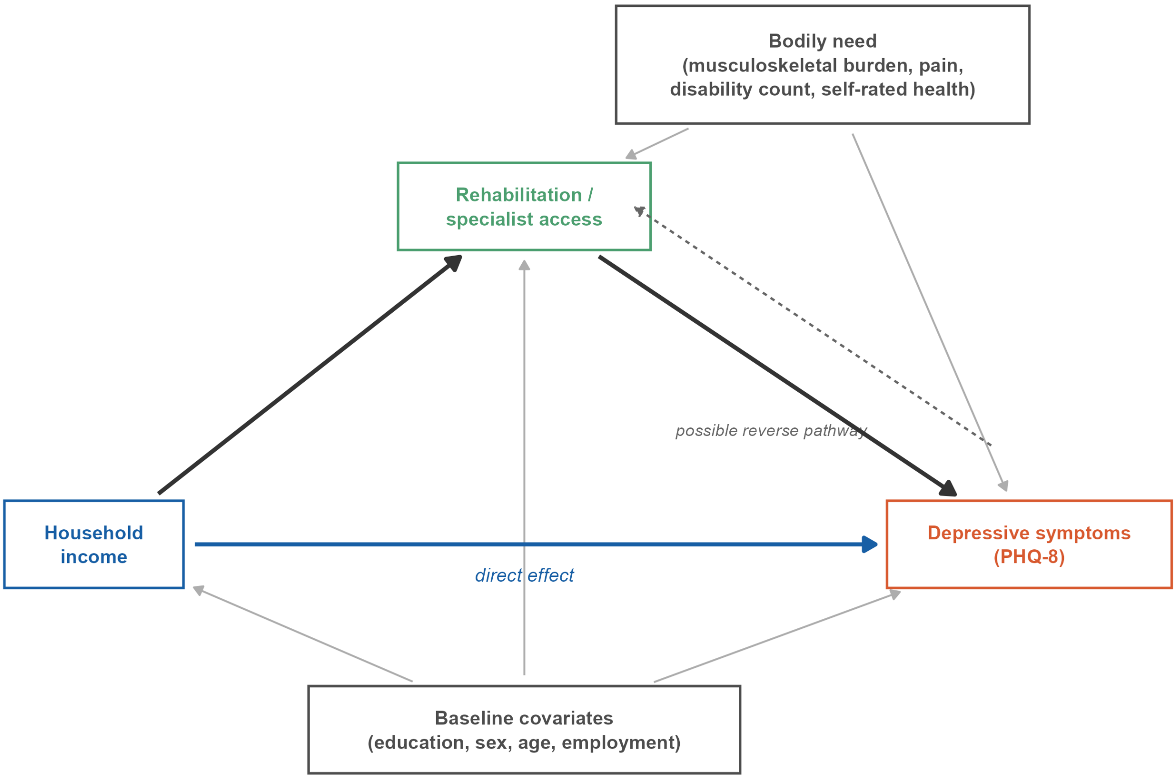

Figure S2. Directed acyclic graph of the assumed pathway from household income through rehabilitation access to depressive symptoms. Household income is the exposure, rehabilitation or specialist access the mediator, and PHQ-8 depressive symptoms the outcome. Bodily need, comprising musculoskeletal burden, pain, disability-domain count, and self-rated health, is modeled as a confounder of the access–depression relationship, and education, sex, age, and employment status enter as baseline confounders of all three variables. The arrow between access and depression is drawn as bidirectional to acknowledge possible confounding by indication inherent in the cross-sectional design.

Table S18. Natural-effect model results (medflex package): full parameter estimates and natural direct and indirect effects (N = 22,740; unweighted; non-parametric bootstrap standard errors).

Parameter	Estimate (log-odds)	SE	z	p	OR [95% CI]
**NATURAL DIRECT AND INDIRECT EFFECTS**					
Natural direct effect (NDE; income→depression, access fixed)	−0.0258	0.0071	−3.655	<0.001	0.975 [0.961–0.988]
Natural indirect effect (NIE; income→access→depression)	+0.0012	0.0004	+2.744	0.006	1.001 [1.000–1.002]

Total effect	−0.0246	0.0070	−3.505	<0.001	0.976 [0.962–0.989]
Mediation ratio (NIE / TE, log-odds scale)	—	—	—	—	−4.8%
**FULL MODEL COVARIATES (log-odds; confounders of access–depression pathway)**					
Self-rated health (GENEL_SAGLIK_SKOR)	+0.5818	0.0525	+11.08	<0.001	1.789
Pain score (AGRI_SKOR)	+0.4719	0.0295	+16.00	<0.001	1.603
Disability-domain count (ENGEL_SAYI)	+0.3002	0.0230	+13.03	<0.001	1.350
Musculoskeletal burden (KASISKELET_YUK)	+0.2089	0.0412	+5.06	<0.001	1.232
Age (YAS)	−0.0244	0.0025	−9.93	<0.001	0.976
Education (EGITIM_DUZEY)	+0.0741	0.0486	+1.53	0.127	1.077
Not employed (CALISMA_FCalismiyor)	+0.0841	0.0944	+0.89	0.373	1.088
Female (CINSIYETKADIN)	+0.0304	0.0855	+0.36	0.722	1.031
Intercept	−4.8337	0.2399	−20.15	<0.001	0.008

Although the NIE achieves statistical significance (p = 0.006) in the unweighted medflex specification owing to the very large sample (N = 22,740), its OR of 1.001 corresponds to an approximate 1.02-fold odds change across the full income range of 20 bands — a magnitude that is clinically and epidemiologically negligible. The second estimator places the indirect effect on the same side of the null but well within sampling noise (CMAverse pure NIE OR = 1.003, 95% CI 0.999–1.008, p = 0.30; Table S19), and the medflex 95% CI (1.0003–1.002) spans a range of trivial magnitudes. The null mediation conclusion therefore rests on the negligible magnitude of the effect under both estimators, not on any single p-value threshold. Natural-effect models were estimated without survey weights, as the medflex implementation does not accommodate design-based weighting; this is noted as a limitation in the main text.

Table S19. CMAverse full causal mediation decomposition: regression-based approach with exposure–mediator interaction term (N = 22,740; bootstrap confidence intervals, 1000 resamples).

Effect	Estimate (OR)	SE	95% CI Lower	95% CI Upper	p / Interpretation
**PRIMARY EFFECTS (odds ratio scale)**					
Controlled direct effect (Rcde)	0.978	0.012	0.956	1.002	p = 0.062 (marginal)
**Pure natural direct effect (Rpnde) ✓**	**0.979**	**0.006**	**0.967**	**0.990**	**p < 0.001 — income protective direct**
Total natural direct effect (Rtnde)	0.979	0.006	0.967	0.990	p < 0.001
**Pure natural indirect effect (Rpnie) ✓**	**1.003**	**0.002**	**0.999**	**1.008**	**p = 0.296 — NULL mediation**
Total natural indirect effect (Rtnie)	1.003	0.002	0.999	1.008	p = 0.282
Total effect (Rte)	0.982	0.006	0.968	0.993	p < 0.001
**EXCESS RELATIVE RISK DECOMPOSITION**					
ERcde (controlled direct)	−0.0170	0.0110	−0.040	+0.001	p = 0.062
ERintref (reference interaction)	−0.0043	0.0089	−0.021	+0.015	p = 0.622 (ns)

ERintmed (mediated interaction)	−0.0001	0.0001	−0.000	+0.000	p = 0.682 (ns)
ERpnie (pure natural indirect)	+0.0028	0.0023	−0.001	+0.008	p = 0.296 (ns)
**PROPORTION SUMMARY**					
Proportion mediated (pm)	−15.2%	33.8	−77.6%	+5.7%	p = 0.282 — wide CI spans null
Proportion attributable to interaction (int)	+23.6%	38.6	−72.4%	+180.9%	p = 0.622
Proportion eliminated (pe)	+8.2%	3.98	−78.0%	+117.5%	p = 0.796
**EXPOSURE–MEDIATOR INTERACTION TERM (outcome model)**					
Income × access interaction coefficient	0.000297	0.01525	—	—	p = 0.985 — no interaction
Access main effect (erisim)	0.3900	0.2094	—	—	p = 0.063 (marginal; confounding by indication)
Income main effect in outcome model	−0.0261	0.01392	—	—	p = 0.061

✓ *Primary effect estimates. The exposure–mediator interaction term (income × access) is negligibly small and non-significant (p = 0.985), confirming no effect modification. The positive, borderline-significant main effect of access on depression (OR ≈ 1.48, p = 0.063 after full adjustment) is consistent with confounding by indication: individuals who access specialist care disproportionately include those with more severe and treatment-resistant conditions, not those who benefit from rehabilitation. Both the proportion mediated (−15%) and its very wide confidence interval (−78% to +6%) confirm that the indirect pathway carries no reliable information about mediation. The mediator regression (income → access): income coefficient = 0.0323, p < 0.001, confirming the relevant confounders were correctly included.*

Table S20. E-value sensitivity analysis for unmeasured confounding, and continuous PHQ-8 sensitivity analysis.

Effect	RR estimate	E-value (est.)	E-value (CI)	Interpretation
**E-VALUE RESULTS (CMAverse; risk/rate ratio scale)**				
Controlled direct effect (Rcde)	0.978	1.175	NA (CI crosses null)	Unmeasured confounder would need RR ≥ 1.17 for both exposure and outcome to explain away the CDE
Pure natural direct effect (Rpnde)	0.979	1.171	1.113	A confounder with RR ≥ 1.17 for both arms could push the lower CI to null — but the effect is robust to moderate confounding
Total natural direct effect (Rtnde)	0.979	1.171	1.112	Essentially identical to Rpnde
Pure natural indirect effect (Rpnie)	1.003	1.056	1.000	E-value lower CI = 1.000; the NIE confidence interval already touches null — no unmeasured confounding needed to eliminate it
Total effect (Rte)	0.982	1.149	1.085	Moderate robustness to confounding
**CONTINUOUS PHQ-8 SENSITIVITY (medflex; outcome = PHQ-8 sum score 0–24)**				
NDE (income → PHQ directly)	−0.0452 (log-linear scale)	SE 0.0049	z = −9.155, p < 0.001	Substantial, significant direct protective effect of income

NIE (income → access → PHQ)	+0.0016 (log-linear scale)	SE 0.0003	z = +4.911, $p < 0.001$	Statistically significant but magnitude = trivial (≈ 0.03 PHQ points across full income range)
Interpretation	—	—	—	Pattern mirrors binary outcome: large direct effect, negligible indirect effect. NIE magnitude non-meaningful despite significance in large sample.

E-values quantify the minimum strength of association that an unmeasured confounder would need to have with both the exposure and the outcome on the risk-ratio scale to fully explain away the observed effect [13,14]. An E-value of 1.17 for the NDE implies that only a confounder with RR ≥ 1.17 for both income and depression would be needed to eliminate the direct effect — a relatively modest threshold reflecting the modest size of the direct OR itself (0.975). For the NIE, the E-value lower CI reaches 1.00, meaning that a confounder of even trivial strength would suffice to account for the already-null indirect effect, further confirming the null mediation conclusion. Continuous PHQ-8 specification: NDE covers full income range (20 × −0.0452 ≈ −0.9 PHQ points across the income distribution); NIE over full range ≈ 20 × 0.0016 ≈ 0.03 PHQ points — epidemiologically negligible.

Section S9: Statistical Software and Reproducibility

All analyses were conducted in R version 4.6.0. A fixed random seed (set.seed(2022)) was specified before all stochastic procedures (bootstrap resampling for medflex and CMAverse, mclust profile enumeration, ranger random forest) to ensure reproducibility. Table S21 lists all packages used, with versions and purposes.

Table S21. R packages used for statistical analysis.

Package	Version	Purpose
survey [28]	4.4	Survey design objects, svyglm, Rao–Scott chi-square, svychisq
lavaan [37]	0.6-21	Confirmatory factor analysis (WLSMV estimator)
psych [38]	2.6.5	Cronbach α, McDonald ω, item-total correlations
mclust [25]	6.1.1	Gaussian finite-mixture models (LPA); BIC, entropy, AvePP
rineq [39]	0.3.0	Concentration index computation and decomposition (cross-validation)
lme4 [40]	1.1-37	Two-level logistic models for MAIHDA (VPC, PCV)
car [41]	3.1-3	Generalized variance inflation factors (GVIF)
splines / stats (R base)	4.6.0	Restricted cubic splines (ns()); regTermTest via survey package
ranger [42]	0.16.0	Probability random forest; permutation variable importance
pdp [43]	0.8.1	Partial-dependence profiles from ranger random forest
medflex [35]	0.6-10	Natural-effect models for mediation (NDE, NIE); bootstrap SE
CMAverse [36]	0.1.0	Regression-based causal mediation; E-value sensitivity analysis
ggplot2 [44]	3.5.1	Figures: LPA profile plot, concentration curve, MAIHDA strata plot, PDP, RCS comparison
dplyr / tidyr / tidyverse	2.0+	Data wrangling and transformation throughout analytic chain
patchwork [45]	1.3.0	Multi-panel figure composition

The microdata from the 2022 Türkiye Health Survey are owned by TurkStat and were accessed under a formal data-use agreement that does not permit public redistribution. All aggregate outputs, model coefficients, and cross-tabulations underlying the reported findings are provided in this Supplementary Document and in the main manuscript tables. Analytic code is available from the corresponding author (M.E. Güvey, emre.guvey@acibadem.edu.tr) upon reasonable request.

References for Supplementary Material

Reference numbers correspond to the reference list of the main manuscript. The numbering is therefore not consecutive: only the references cited in this Supplementary Material are listed below, and each of them also appears in the main manuscript reference list.

2. Wagstaff, A.; van Doorslaer, E.; Watanabe, N. On Decomposing the Causes of Health Sector Inequalities with an Application to Malnutrition Inequalities in Vietnam. J. Econom. 2003, 112, 207–223. https://doi.org/10.1016/S0304-4076(02)00161-6.
3. O'Donnell, O.; van Doorslaer, E.; Wagstaff, A.; Lindelow, M. Analyzing Health Equity Using Household Survey Data; World Bank: Washington, DC, USA, 2008.
13. VanderWeele, T.J. Explanation in Causal Inference: Methods for Mediation and Interaction; Oxford University Press: New York, NY, USA, 2015; ISBN 9780199325870.
14. VanderWeele, T.J.; Ding, P. Sensitivity Analysis in Observational Research: Introducing the E-Value. Ann. Intern. Med. 2017, 167, 268–274. https://doi.org/10.7326/M16-2607.
15. Turkish Statistical Institute. Türkiye Health Survey, 2022; Turkish Statistical Institute: Ankara, Türkiye, 2022. Available online: https://data.tuik.gov.tr/Bulten/Index?p=Turkiye-Saglik-Arastirmasi-2022-49747 (accessed on 27 December 2025).
16. Kroenke, K.; Strine, T.W.; Spitzer, R.L.; Williams, J.B.W.; Berry, J.T.; Mokdad, A.H. The PHQ-8 as a Measure of Current Depression in the General Population. J. Affect. Disord. 2009, 114, 163–173. https://doi.org/10.1016/j.jad.2008.06.026.
17. Madans, J.H.; Loeb, M.E.; Altman, B.M. Measuring Disability and Monitoring the UN Convention on the Rights of Persons with Disabilities: The Work of the Washington Group on Disability Statistics. BMC Public Health 2011, 11, S4. https://doi.org/10.1186/1471-2458-11-S4-S4.
19. Kenny, D.A.; Kaniskan, B.; McCoach, D.B. The Performance of RMSEA in Models With Small Degrees of Freedom. Sociol. Methods Res. 2015, 44, 486–507. https://doi.org/10.1177/0049124114543236.
20. Wu, H.; Estabrook, R. Identification of Confirmatory Factor Analysis Models of Different Levels of Invariance for Ordered Categorical Outcomes. Psychometrika 2016, 81, 1014–1045. https://doi.org/10.1007/s11336-016-9506-0.
21. Chen, F.F. Sensitivity of Goodness of Fit Indexes to Lack of Measurement Invariance. Struct. Equ. Model. 2007, 14, 464–504. https://doi.org/10.1080/10705510701301834.
22. Cheung, G.W.; Rensvold, R.B. Evaluating Goodness-of-Fit Indexes for Testing Measurement Invariance. Struct. Equ. Model. 2002, 9, 233–255. https://doi.org/10.1207/S15328007SEM0902_5.
25. Scrucca, L.; Fop, M.; Murphy, T.B.; Raftery, A.E. Mclust 5: Clustering, Classification and Density Estimation Using Gaussian Finite Mixture Models. R J. 2016, 8, 289–317. https://doi.org/10.32614/RJ-2016-021.
28. Lumley, T. Analysis of Complex Survey Samples. J. Stat. Softw. 2004, 9, 1–19. https://doi.org/10.18637/jss.v009.i08.
26. Vermunt, J.K. Latent Class Modeling with Covariates: Two Improved Three-Step Approaches. Polit. Anal. 2010, 18, 450–469. https://doi.org/10.1093/pan/mpq025.
27. Bandeen-Roche, K.; Miglioretti, D.L.; Zeger, S.L.; Rathouz, P.J. Latent Variable Regression for Multiple Discrete Outcomes. J. Am. Stat. Assoc. 1997, 92, 1375–1386. https://doi.org/10.1080/01621459.1997.10473658.
30. Evans, C.R.; Williams, D.R.; Onnela, J.-P.; Subramanian, S.V. A Multilevel Approach to Modeling Health Inequalities at the Intersection of Multiple Social Identities. Soc. Sci. Med. 2018, 203, 64–73. https://doi.org/10.1016/j.socscimed.2017.11.011.
31. Merlo, J. Multilevel Analysis of Individual Heterogeneity and Discriminatory Accuracy (MAIHDA) within an Intersectional Framework. Soc. Sci. Med. 2018, 203, 74–80. https://doi.org/10.1016/j.socscimed.2017.12.026.
32. Evans, C.R.; Borrell, L.N.; Bell, A.; Holman, D.; Subramanian, S.V.; Leckie, G. Clarifications on the Intersectional MAIHDA Approach: A Conceptual Guide and Response to Wilkes and Karimi (2024). Soc. Sci. Med. 2024, 350, 116898. https://doi.org/10.1016/j.socscimed.2024.116898.
42. Wright, M.N.; Ziegler, A. Ranger: A Fast Implementation of Random Forests for High Dimensional Data in C++ and R. J. Stat. Softw. 2017, 77, 1–17. https://doi.org/10.18637/jss.v077.i01.
35. Steen, J.; Loeys, T.; Moerkerke, B.; Vansteelandt, S. Medflex: An R Package for Flexible Mediation Analysis Using Natural Effect Models. J. Stat. Softw. 2017, 76. https://doi.org/10.18637/jss.v076.i11.
36. Shi, B.; Choirat, C.; Coull, B.A.; VanderWeele, T.J.; Valeri, L. CMAverse: A Suite of Functions for Reproducible Causal Mediation Analyses. Epidemiology 2021, 32, e20–e22. https://doi.org/10.1097/EDE.0000000000001378.

37. Rosseel, Y. Lavaan: An R Package for Structural Equation Modeling. J. Stat. Softw. 2012, 48, 1–36. https://doi.org/10.18637/jss.v048.i02.
38. Revelle, W. Psych: Procedures for Psychological, Psychometric, and Personality Research, version 2.5.6; Northwestern University: Evanston, IL, USA, 2025. Available online: https://CRAN.R-project.org/package=psych (accessed on 27 December 2025).
39. Meili, K.W. Rineq: Concentration Index and Decomposition for Health Inequalities, version 0.3.0; 2025. Available online: https://CRAN.R-project.org/package=rineq (accessed on 27 December 2025).
40. Bates, D.; Mächler, M.; Bolker, B.; Walker, S. Fitting Linear Mixed-Effects Models Using Lme4. J. Stat. Softw. 2015, 67, 1–48. https://doi.org/10.18637/jss.v067.i01.
41. Fox, J.; Weisberg, S. Car: Companion to Applied Regression, version 3.1-3; 2024. Available online: https://CRAN.R-project.org/package=car (accessed on 27 December 2025).
43. Greenwell, B.M. Pdp: An R Package for Constructing Partial Dependence Plots. R J. 2017, 9, 421–436. https://doi.org/10.32614/RJ-2017-016.
44. Wickham, H. Ggplot2: Elegant Graphics for Data Analysis; Springer: New York, NY, USA, 2016.
45. Pedersen, T.L. Patchwork: The Composer of Plots, version 1.3.0; 2024. Available online: https://CRAN.R-project.org/package=patchwork (accessed on 27 December 2025).
